# From cN2 to ypN0: Nodal Downstaging After Neoadjuvant Treatment in Breast Cancer—A Retrospective Cohort Study from a Tertiary Cancer Center

**DOI:** 10.3390/medsci14060642

**Published:** 2026-10-06

**Authors:** Vlad Alexandru Gâta, Radu Alexandru Ilieș, Ana Maria Mureșan-Bădescu, Teodor Marian Vancea, Anda Gâta, Nicoleta Zenovia Antone, Roxana Pintican, Simona Ioana Filip, Gerald Gheorghe Filip, Eugeniu Darii, Iyad Shahin, Răzvan-Valentin Scăunașu, Maximilian Vlad Muntean

**Affiliations:** 1 Department of Oncological Surgery, “Iuliu Hațieganu” University of Medicine and Pharmacy, 400012 Cluj-Napoca, Romania; 2 Department of Oncological Surgery and Gynecological Oncology, “Prof. Dr. Ion Chiricuță” Institute of Oncology, 400015 Cluj-Napoca, Romania; 3 Faculty of Medicine, “Iuliu Hațieganu” University of Medicine and Pharmacy, 400012 Cluj-Napoca, Romania; 4 Department of Otorhinolaryngology, “Iuliu Hațieganu” University of Medicine and Pharmacy, 400012 Cluj-Napoca, Romania; 5 Department of Oncology, “Prof. Dr. Ion Chiricuță” Institute of Oncology, 400015 Cluj-Napoca, Romania; 6 Department of Radiology, “Iuliu Hațieganu” University of Medicine and Pharmacy, 400012 Cluj-Napoca, Romania; 7 Department of Surgery, Regina Maria Ponderas Academic Hospital, 014142 Bucharest, Romania; 8 Regina Maria Hospital, 500091 Brașov, Romania; 9 Ovidius Clinical Hospital, 905900 Constanța, Romania; 10 “Carol Davila” University of Medicine and Pharmacy, 050474 Bucharest, Romania; 11 Department of General Surgery, “Colțea” Clinical Hospital, 030171 Bucharest, Romania; 12 Department of Plastic Surgery, “Prof. Dr. Ion Chiricuță” Institute of Oncology, 400015 Cluj-Napoca, Romania; 13 Department of Plastic and Reconstructive Surgery, “Iuliu Hațieganu” University of Medicine and Pharmacy, 400012 Cluj-Napoca, Romania

**Keywords:** cN2 breast cancer, neoadjuvant treatment, axillary response, molecular subtype, histological grade, TILs, axillary nodal clearance

## Abstract

**Background/Objectives**: In patients with clinically node-positive breast cancer, axillary response to neoadjuvant systemic treatment is heterogeneous and might depend on tumor biology. In patients with cN2 disease, the identification of factors linked to nodal response could improve risk stratification and contribute to the development of individualized post-neoadjuvant axillary management strategies. **Methods**: This cohort study has a retrospective design and included 560 female patients with invasive breast cancer (proven via biopsy) and cN2 disease. All patients received neoadjuvant systemic treatment accompanied by breast and axillary surgery at the Institute of Oncology “Prof. Dr. Ion Chiricuță” Cluj-Napoca (IOCN), Romania, between 2017 and 2025. The primary endpoint was the rate of axillary pathological complete response (pCR) following neoadjuvant systemic therapy, defined as ypN0. Secondary endpoints included breast pCR (ypT0), overall pCR (ypT0N0), the association between breast and axillary pathological response and survival analysis, including overall survival (OS) and disease-free survival (DFS). Clinicopathological predictors were assessed using univariate and multivariate logistic regression, while survival outcomes were evaluated with time-to-event analyses. **Results**: Complete axillary pathological response occurred in 282/560 patients (50.4%), and it increased to 53.2% when ypN0, including ypN0(i+). Breast pathological complete response was achieved in 31.2%, while overall pCR was 27.0%. Axillary response was significantly different in terms of molecular subtype, as ypN0 rates were 26.6% in Luminal A-like, 32.5% in Luminal B-like, 78.9% in HER2-positive and 72.6% in TNBC (*p* < 0.001). Nottingham grade 3 and HER2-positive disease were independently related to increased odds of ypN0, while TILs <10% were associated with lower odds of complete axillary response when multivariate analysis was performed. Breast and axillary responses were significantly associated (but were not interchangeable), as ypT0 showed 53.5% sensitivity and 91.9% specificity for ypN0. Overall pCR was associated with improved DFS (*p* = 0.040), while ypN0 and ypT0 were not significantly associated with DFS. No significant OS differences were noticed regarding pathological response. **Conclusions**: Nearly half of patients with cN2 breast cancer presented a complete axillary response after the completion of neoadjuvant treatment. Molecular subtype, histological grade and TILs were strongly associated with axillary response. In our cohort, breast pathological response alone was insufficient in order to be regarded as a predictor of nodal clearance, further supporting independent assessments of breast and axillary response. Further prospective studies are needed to determine whether these findings can inform individualized axillary management after neoadjuvant treatment.

## 1. Introduction

Neoadjuvant systemic therapy represents a standard treatment strategy for patients presenting with locally advanced and clinically node-positive breast cancer and allows for the assessment of treatment response in both the breast and axilla [1]. The likelihood of obtaining an axillary pathological complete response (pCR) depends on tumor subtype, as the highest reported rates occur in HER2-positive and triple-negative breast cancer (TNBC), whereas in hormone receptor-positive/HER2-negative disease, the rates are significantly lower [1].

Baseline nodal burden also influences axillary response. A higher clinical nodal stage implies a lower probability of achieving ypN0 across different subtypes of breast cancer, highlighting that initial axillary disease extent remains relevant even in tumors with an increased biological sensitivity to systemic therapy [2]. This is particularly relevant in cases with cN2 disease, in whom the initial axillary tumor burden is greater than in lower clinical nodal stages.

Despite the advanced nodal presentation, a significant proportion of patients with cN2/cN3 disease achieve nodal downstaging after completing neoadjuvant systemic therapy [3].

Despite breast and axillary pathological responses being closely related, their association is not absolute. In initially node-positive breast cancer, breast pCR is linked to a higher probability of axillary clearance [4]. The CALGB 40601 and 40603 study indicated that pretreatment clinical nodal status and breast response are the main predictors of ypN0 in TNBC and HER2-positive molecular subtypes [5].

However, the concordance between breast and axillary response decreases in parallel with the increase in nodal burden [6]. This relationship has relevance in cN2 disease, where breast pathological complete response has been strongly linked to axillary clearance following neoadjuvant therapy [7].

Axillary pathological response also has prognostic value. In initially node-positive breast cancer, axillary pCR has been linked to improved disease-free survival, while it has been found that patients with persistent nodal disease following neoadjuvant treatment have worse outcomes [8,9]. Long-term data indicate that cases converting from node-positive to ypN0 might still have worse outcomes in comparison with cases who were node-negative at diagnosis, highlighting that pathological nodal clearance does not fully eliminate the prognostic significance of the initial disease burden [10].

An essential component in the management of breast cancer is represented by accurate axillary staging (particularly in the setting of neoadjuvant treatment), and sentinel lymph node biopsy has become an approach for axillary assessment, with different techniques being available for identifying the sentinel lymph node [11,12,13,14,15].

Even though recent studies have specifically evaluated cN2/cN3 disease, the available body of evidence remains limited in comparison with the broader clinically node-positive population.

The primary endpoint of this study was to evaluate the axillary response to neoadjuvant systemic therapy in women with cN2 breast cancer and to determine the rate of conversion to pathologically node-negative disease (ypN0) following neoadjuvant treatment and surgery. Secondary objectives included assessing the association between axillary response and clinicopathological and biological characteristics, identifying predictors of complete axillary response, evaluating the relationship between breast and axillary pathological response, and exploring the association between pathological response and survival outcomes.

## 2. Materials and Methods

### 2.1. Study Design and Setting

This retrospective cohort study was conducted at the Institute of Oncology Cluj-Napoca (IOCN), Romania. The study period extended from 2017 to 2025. Patients with primary invasive breast cancer who received neoadjuvant systemic treatment followed by breast surgery and surgical axillary evaluation at IOCN were included in the study.

The study population was defined according to the prespecified eligibility criteria. Patients had biopsy-proven invasive breast carcinoma and cN2 nodal disease at initial evaluation, established by clinical and imaging assessment or histological confirmation of nodal involvement, according to institutional practice.

### 2.2. Eligibility Criteria

The eligible patients fulfilled all prespecified inclusion criteria:

- Female sex;
- Primary breast cancer treated at IOCN;
- Histopathologically confirmed invasive breast carcinoma;
- cN2 disease at initial diagnosis;
- Completion of neoadjuvant systemic treatment at IOCN;
- Breast surgery, either breast-conserving surgery or mastectomy, combined with surgical axillary assessment;
- Availability of the clinical, imaging and pathological information required for the analyses;
- Completion of neoadjuvant treatment followed by surgery at IOCN without loss to follow-up before surgery.

Patients excluded from the study fulfilled at least one of the following criteria:

- Presence of de novo metastatic disease (cM1);
- Primary surgery without neoadjuvant systemic treatment;
- Incomplete or abandoned neoadjuvant treatment;
- Absence of surgical axillary evaluation;
- Another active malignancy or synchronous malignancy that could affect survival and interpretation of the study outcomes;
- Pure carcinoma in situ without an invasive component;
- Missing key clinical, treatment or pathological information, including unknown pN status or an incomplete pathological report.

Non-melanoma skin cancer may be considered an exception to the exclusion criteria for previous malignancy, according to the final study protocol.

The necessary data were extracted from the institutional database and included in the study if the patients fulfilled the predefined eligibility criteria. The total cohort included patients who underwent neoadjuvant systemic therapy and breast and axillary surgery at IOCN. Among these, Cohort A consisted of patients who completed their entire treatment pathway at our institution, meaning both neoadjuvant and adjuvant treatment. The other 227 patients underwent only neoadjuvant treatment and surgery at IOCN. The adjuvant treatment was received at another institution. These patients were not lost to follow-up and continued to attend regular follow-up visits.

### 2.3. Clinical and Tumor Characteristics

Baseline clinical and tumor variables consisted of patient age at diagnosis, menstrual status, clinical stage, clinical tumor category (cT), molecular subtype, estrogen receptor/progesterone receptor (ER/PR) status, histological subtype, Nottingham histological grade, Ki-67, tumor-infiltrating lymphocytes (TILs), and associated ductal carcinoma in situ (DCIS).

Molecular subtypes were classified as Luminal A-like, Luminal B-like, HER2-positive and TNBC, based on immunohistochemical characteristics, including ER, PR, HER2 and Ki-67 expression, together with histological grade using the Nottingham grading system (in accordance with the St. Gallen International Expert Consensus on the Primary Therapy of Early Breast Cancer 2013) [16].

Clinical nodal staging was performed according to the 8th edition of the American Joint Committee on Cancer (AJCC) Cancer Staging [17,18]. For the purposes of the current study, cN2 refers to patients with clinically advanced ipsilateral axillary nodal disease, while ypN0 denotes the absence of residual nodal metastases on pathological examination after neoadjuvant systemic therapy. Moreover, cN2 disease was analyzed as a single category (instead of being further subdivided into cN2a and cN2b), aiming to simplify the analysis and maintain consistency with the staging information recorded in the institutional database. Clinical N2 status was established based on the clinical and imaging assessment performed at diagnosis, according to institutional practice. The database did not systematically distinguish between cN2a and cN2b subcategories.

### 2.4. Neoadjuvant Treatment

Neoadjuvant treatment consisted of chemotherapy or endocrine therapy according to the available study data. The type of neoadjuvant systemic treatment was included among the variables evaluated for associations with pathological response.

Neoadjuvant systemic treatment was selected by the institutional multidisciplinary team based on tumor subtype, stage, patient-related factors, and the treatment recommendations and institutional protocols that were applicable at the time of diagnosis.

As these recommendations evolved during the study period, treatment regimens varied according to calendar year.

### 2.5. Surgical Treatment and Pathological Assessment

Patients underwent breast surgery which consisted of either mastectomy or breast-conserving surgery (lumpectomy). Surgical axillary evaluation was mandatory for study eligibility, with axillary lymph node dissection (ALND) specified in the study protocol.

According to the institutional protocol, axillary nodal involvement was confirmed based on histopathological examination of the largest clinically or radiologically suspicious node. All patients included in the study underwent ALND as part of the surgical management.

Pathological variables included pathological tumor stage (ypT), pathological nodal status (ypN), number of excised lymph nodes, number of positive lymph nodes, resection margins, breast pathological complete response (ypT0), axillary pathological complete response (ypN0), and overall pCR (ypT0N0).

Pathological axillary response was classified according to the AJCC 8th edition staging system [17].

Breast pCR was defined as ypT0, indicating complete absence of residual invasive carcinoma and ductal carcinoma in situ in the breast specimen. Thus, residual DCIS was not considered compatible with breast pathological complete response.

Isolated tumor cells were classified as ypN0(i+), while micrometastatic disease was classified as ypN1mi. For the primary analysis, axillary pathological complete response was defined as conventional ypN0. Patients with ypN0(i+) were analyzed separately as an additional category and were not included in the conventional ypN0 endpoint.

Overall pathological complete response was defined as ypT0N0, requiring complete absence of residual invasive carcinoma and ductal carcinoma in situ in the breast and absence of residual nodal metastases.

### 2.6. Follow-Up

Patients were followed according to the institutional follow-up schedule, with regular assessments at approximately 3, 6, 12, 18, 24, 36, 48, and 60 months after completion of the treatment. For the purposes of the present study, follow-up data were collected up to 1 June 2026. For survival analyses, Cohort A included patients treated between 2017 and 2022 who completed both neoadjuvant and adjuvant treatment at IOCN and had a minimum follow-up of 36 months, providing an adequate observation period for the assessment of overall survival and disease-free survival.

### 2.7. Study Outcomes

The principal outcome was to achieve axillary pCR, defined as ypN0 after neoadjuvant systemic treatment.

Secondary pathological outcomes evaluated breast pathological complete response (ypT0), overall pCR (ypT0N0), and the relationship between breast and axillary pathological response.

In addition, available survival outcomes included overall survival and disease-free survival, with median follow-up detailed in Section 3. Overall survival was defined as the time from diagnosis to death from any cause, whereas disease-free survival was defined as the time from diagnosis to documented disease recurrence or death.

The total cohort was used for descriptive and pathological response analyses, while survival analyses were restricted to Cohort A to ensure consistency and completeness of time-to-event data.

### 2.8. Statistical Analysis

Categorical variables were summarized as absolute numbers and percentages. Continuous variables were summarized using the median and interquartile range (IQR), as reported in the study database.

Associations between categorical variables and pathological response were assessed using the chi-square test. Odds ratios (ORs) with 95% confidence intervals (CIs) were reported for associations between candidate predictors and pathological response.

Logistic regression analyses were used to evaluate predictors of complete axillary response, breast complete response, and overall pCR. Both crude and adjusted ORs with 95% CIs were reported. The multivariate models included the variables specified in the study analysis. Because no Luminal A-like tumor achieved ypT0N0, the ypT0N0 model was estimated using Firth’s penalized-likelihood logistic regression. Subtype contrasts relative to Luminal A-like were not estimable and are not reported.

The relationship between breast and axillary response was evaluated using crude and adjusted logistic regression, as well as measures of concordance, including Cohen’s kappa. The diagnostic performance of ypT0 for predicting ypN0 was assessed by sensitivity, specificity, positive predictive value and negative predictive value.

Overall survival and disease-free survival were evaluated using time-to-event analyses, with comparisons between groups performed using the log-rank test.

A two-sided *p* value of <0.05 was considered statistically significant, according to the statistical analyses reported in the study results.

All statistical analyses were performed using Python version 3.12 (Python Software Foundation, Wilmington, DE, USA). Data processing and manipulation were performed using pandas (version 2.2.2) and NumPy (version 2.0.2), while statistical analyses were conducted using SciPy (version 1.16.3). Survival analyses were performed using lifelines (version 0.30.3) and graphical representations were generated using Matplotlib (version 3.10.0).

### 2.9. Ethical Considerations

This study was approved by the Ethics Committee of the Institute of Oncology “Prof. Dr. Ion Chiricuță” Cluj-Napoca (Ethics Committee Evaluation Report No. 393/22 June 2026; Application No. 6207/18 June 2026).

Through the preparation of this manuscript, the large language model ChatGPT (GPT-5.6 Luna, OpenAI, San Francisco, CA, USA) was used for language editing issues, including grammar, spelling, clarity and English-language refinement. The authors are fully responsible for the content of the manuscript, including the accuracy and integrity of the data, analyses, interpretations and conclusions. All data presented in this manuscript are original and were verified by the authors. All AI-assisted edits and suggestions were carefully reviewed and verified by the authors, who retain full responsibility for the final version of the manuscript.

## 3. Results

### 3.1. Study Population and Baseline Characteristics

The overall study cohort comprised a total of 560 patients, while the subgroup designated as Cohort A included 333 patients treated and followed at IOCN (Table 1).

**Figure 1:**
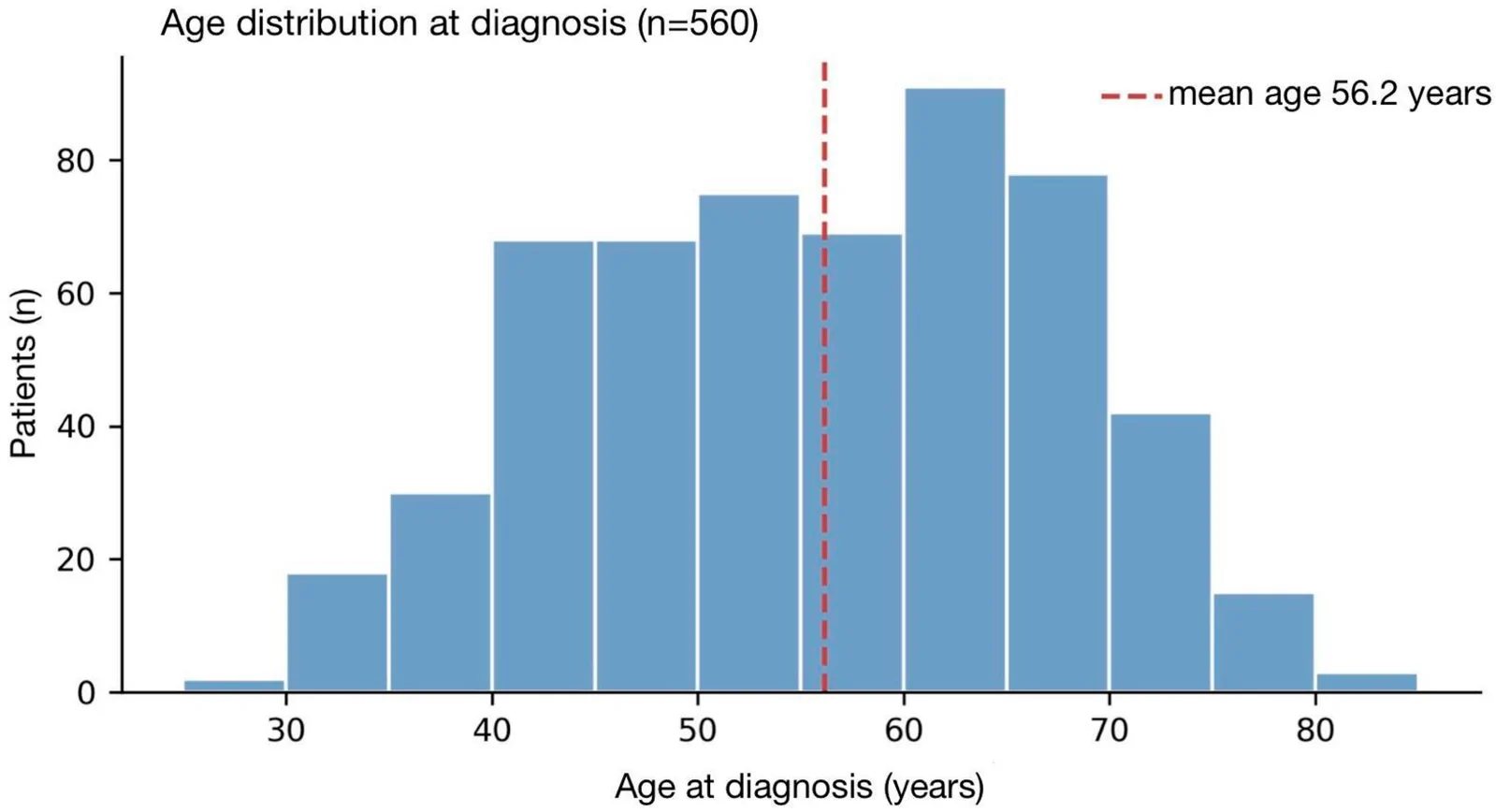
Distribution of patients according to age at diagnosis.

The mean age at diagnosis was 56.2 years in the total population (Figure 1) and 56.0 years in Cohort A. Overall, 179 patients (32.0%) were menstruating cyclically and 381 (68.0%) were postmenopausal. The corresponding proportions in Cohort A were 30.6% and 69.4%, respectively.

Most patients had clinical stage IIIA disease (547/560, 97.7%). The remaining comprised 10 patients (1.8%) with stage IIIB, 2 (0.4%) with stage IIB, and 1 (0.2%) with stage III disease with unspecified substage (their inclusion was based on the same baseline clinical staging criteria applied to the remaining patients). The cT category was cT1–2 in 398 patients (71.1%) and cT3–4 in 162 patients (28.9%). In Cohort A, these proportions were 72.7% and 27.3%, respectively.

Invasive ductal carcinoma was the predominant histological subtype, accounting for 516 patients (92.1%), followed by invasive lobular carcinoma in 27 patients (4.8%), and mucinous carcinoma in 15 patients (2.7%). The remaining 2 patients were diagnosed with apocrine carcinoma (0.2%), and cribriform carcinoma (0.2%). Nottingham grade was G1 in 84 patients (15.0%), G2 in 253 (45.2%), and G3 in 223 (39.8%). The median Ki-67 was 25.0% (IQR, 15.0–50.0), while the median percentage of TILs was 10.0% (IQR, 5.0–30.0).

**Figure 2:**
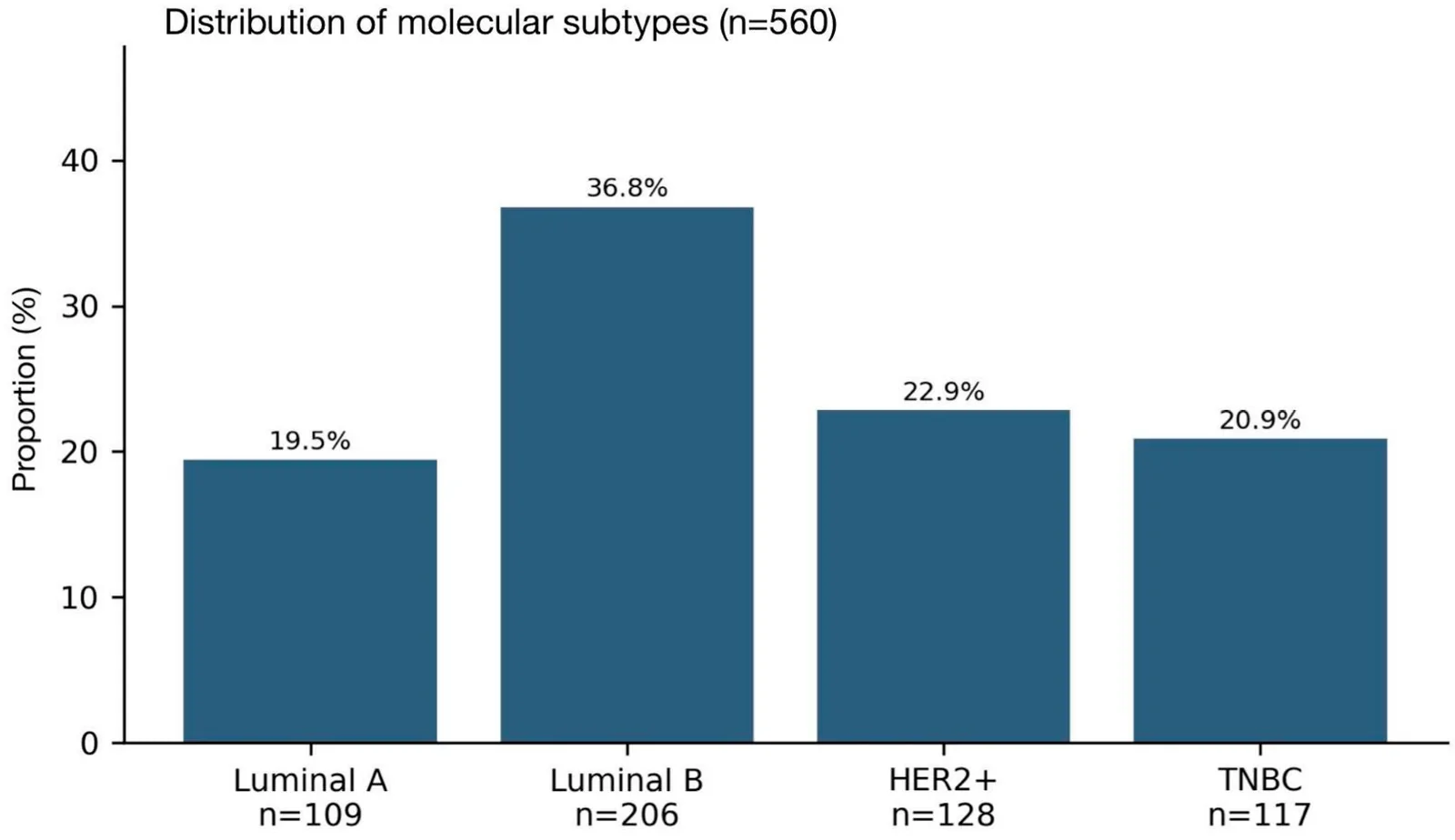
Distribution of molecular subtypes.

Regarding the molecular subtype, the distribution consisted of 109 patients (19.5%) with Luminal A-like, 206 (36.8%) with Luminal B-like, 128 (22.9%) with HER2-positive disease, and 117 (20.9%) with TNBC (Figure 2). ER/PR were positive in 386 patients (68.9%) and negative in 174 (31.1%). The corresponding molecular subtype proportions in Cohort A were 17.1% with Luminal A-like, 36.9% with Luminal B-like, 27.3% with HER2-positive, and 18.6% with TNBC, respectively.

### 3.2. Neoadjuvant and Surgical Treatment

Out of the total 560 patients, neoadjuvant chemotherapy was administered to 506 (90.4%), whereas 54 (9.6%) received endocrine therapy. Breast surgery consisted of mastectomy in 409 patients (73.0%) and breast-conserving surgery in 151 (27.0%). R0 resection was achieved in 555 patients (99.1%), while 5 (0.9%) had an R1 margin. The median number of excised lymph nodes was 15.0 (IQR, 12.0–19.0), and the median number of positive lymph nodes after neoadjuvant treatment was 0.0 (IQR, 0.0–3.0).

### 3.3. Pathological Response

The breast pathological complete response rate (ypT0) was 31.2% (175/560; 95% CI, 27.5–35.2). Complete axillary pathological response rate (ypN0) was 50.4% (282/560; 95% CI, 46.2–54.5). Overall pCR rate (ypT0N0) was 27.0% (151/560; 95% CI, 23.4–30.8). When ypN0 including ypN0(i+) was considered, the rate was 53.2% (298/560; 95% CI, 49.1–57.3). In Cohort A, the corresponding rates were 31.5%, 51.7%, 27.6%, and 55.3%, respectively (Table 2).

Pathological response differed significantly according to molecular subtype (Table 3). The ypN0 rate was 26.6% in Luminal A-like (29/109), 32.5% in Luminal B-like (67/206), 78.9% in HER2-positive disease (101/128), and 72.6% in TNBC (85/117) (chi-square *p* < 0.001). Global ypT0N0 rates were 0% for Luminal A-like, 8.3% for Luminal B-like, 62.5% for HER2-positive disease, and 46.2% for TNBC (*p* < 0.001). The corresponding differences in ypT0 were also statistically significant (*p* < 0.001).

The cT category was not significantly associated with pathological response. Among patients with cT1–2 disease, ypT0, ypN0 and ypT0N0 rates were 33.2%, 50.3%, and 28.6%, respectively, compared with similar results among patients with cT3–4 disease (26.5%, 50.6%, and 22.8%). The corresponding *p* values were 0.152, 1.000, and 0.194 (Table 4).

### 3.4. Predictors of Breast Pathological Complete Response

The breast pathological complete response rate was significantly associated with several clinicopathological characteristics (Table 5). In the total cohort, Nottingham grade 3 was significantly associated with ypT0 (OR, 14.55; 95% CI, 9.35–22.67; *p* < 0.001), whereas Ki-67 <20% (OR, 0.05; 95% CI, 0.02–0.12; *p* < 0.001), TILs <10% (OR, 0.11; 95% CI, 0.07–0.18; *p* < 0.001), and ER/PR positivity (OR, 0.12; 95% CI, 0.08–0.18; *p* < 0.001) were associated with a lower probability of breast complete response. HER2 positivity was significantly associated with ypT0 (OR, 7.14; 95% CI, 4.64–10.97; *p* < 0.001).

In multivariate analysis, Nottingham grade 3 remained independently associated with ypT0 (adjusted OR, 3.68; 95% CI, 2.03–6.64; *p* < 0.001), while Ki-67 <20% (adjusted OR, 0.22; 95% CI, 0.09–0.56; *p* = 0.001) and TILs <10% (adjusted OR, 0.31; 95% CI, 0.17–0.57; *p* < 0.001) were inversely associated with breast complete response (Table 6). HER2-positive disease showed a strong independent association with ypT0 (adjusted OR, 18.59; 95% CI, 4.00–86.48; *p* < 0.001). TNBC was also independently associated with ypT0 in the total cohort (adjusted OR, 5.73; 95% CI, 1.18–27.83; *p* = 0.030). cT3–4 disease was inversely associated with breast complete response (adjusted OR, 0.46; 95% CI, 0.27–0.80; *p* = 0.005).

### 3.5. Predictors of Axillary Pathological Complete Response

In univariate analyses, several clinicopathological factors were associated with ypN0 (Table 7). Nottingham grade 3 was associated with a higher likelihood of ypN0 (OR, 6.68; 95% CI, 4.05–11.02; *p* < 0.001), whereas Ki-67 <20% (OR, 0.26; 95% CI, 0.16–0.43; *p* < 0.001), TILs <10% (OR, 0.13; 95% CI, 0.08–0.21; *p* < 0.001), positive ER/PR (OR, 0.17; 95% CI, 0.10–0.30; *p* < 0.001) showed the reported associations in the subgroup analysis. In the total cohort, the corresponding ORs were 7.24 for Nottingham grade 3, 0.24 for Ki-67 <20%, 0.10 for TILs <10%, 0.18 for positive ER/PR.

HER2 positivity was strongly associated with complete axillary response. In the total cohort, ypN0 occurred in 101/130 HER2-positive patients (77.7%) compared with 181/430 HER2-negative patients (42.1%), corresponding to an OR of 4.79 (95% CI, 3.04–7.55; *p* < 0.001).

In the molecular subtype analysis using Luminal A-like as the reference, the total-cohort OR for ypN0 was 1.33 (95% CI, 0.79–2.23; *p* = 0.278) for Luminal B-like, 10.32 (95% CI, 5.66–18.82; *p* < 0.001) for HER2-positive disease, and 7.33 (95% CI, 4.07–13.19; *p* < 0.001) for TNBC.

In multivariate analysis of the total cohort, Nottingham grade 3 remained independently associated with complete axillary response (adjusted OR, 2.27; 95% CI, 1.31–3.92; *p* = 0.003), as represented in Table 8. TILs <10% was inversely associated with ypN0 (adjusted OR, 0.18; 95% CI, 0.11–0.27; *p* < 0.001), while the association with DCIS <10% did not reach statistical significance (adjusted OR, 1.60; 95% CI, 0.97–2.65; *p* = 0.066). HER2-positive disease remained independently associated with ypN0 (adjusted OR, 3.05; 95% CI, 1.44–6.49; *p* = 0.004), whereas TNBC was not significantly associated with ypN0 in this model (adjusted OR, 1.52; 95% CI, 0.67–3.45; *p* = 0.316). cT3–4 disease was not independently associated with ypN0 (adjusted OR, 0.85; 95% CI, 0.54–1.35; *p* = 0.487).

### 3.6. Relationship Between Breast and Axillary Response

Among the reported cases, residual breast tumor with ypN+ disease was present in 148 patients (44.4%), residual breast tumor with ypN0 in 80 (24.0%), ypT0 with ypN+ in 13 (3.9%), and ypT0 with ypN0 in 92 (27.6%). Thus, complete breast response was associated with complete axillary response, with a crude OR of 13.09 (95% CI, 6.89–24.86; *p* < 0.001), as represented in Table 9. After adjustment for molecular subtype, Nottingham grade, TILs and Ki-67, the association remained significant (adjusted OR, 4.75; 95% CI, 2.24–10.07; *p* < 0.001).

The overall concordance between breast and axillary pathological response was 72.1%, with a Cohen’s kappa of 0.45. Using ypT0 as a predictor of ypN0, sensitivity was 53.5%, specificity 91.9%, positive predictive value 87.6%, and negative predictive value 64.9%. Notably, 13/105 patients (12.4%) had positive axillary disease despite a pCR in the breast, whereas 80/228 patients (35.1%) had ypN0 despite residual breast tumor.

The relationship between breast and axillary response varied according to molecular subtype (Table 10). Among patients with ypT0, ypN0 was observed in 0/2 patients with Luminal A-like, 9/14 (64%) with Luminal B-like, 57/60 (95%) with HER2-positive disease, and 26/29 (90%) with TNBC. Among patients with residual breast tumor, ypN0 was observed in 13/55 (24%) with Luminal A-like, 31/109 (28%) with Luminal B-like, 17/31 (55%) with HER2-positive disease, and 19/33 (58%) with TNBC.

### 3.7. Predictors of Overall pCR

In multivariable analysis of overall pCR (ypT0N0), Nottingham grade 3 was independently related to a higher likelihood of complete pathological response in the total cohort (adjusted OR, 6.40; 95% CI, 3.24–12.67; *p* < 0.001). As shown in Table 11, Ki-67 <20% was associated with a lower probability of obtaining ypT0N0 (adjusted OR, 0.30; 95% CI, 0.10–0.91; *p* = 0.033), as was TILs <10% (adjusted OR, 0.18; 95% CI, 0.08–0.38; *p* < 0.001).

DCIS <10% was independently related to a higher likelihood of overall pCR (adjusted OR, 2.32; 95% CI, 1.14–4.72; *p* = 0.020). Subtype-specific associations could not be estimated in the adjusted model, because no Luminal A-like tumor achieved ypT0N0. Overall pCR rates differed markedly by subtype (Table 3). In comparison with cT1–2 disease, T3–4 disease was independently associated with lower odds of ypT0N0 (adjusted OR, 0.45; 95% CI, 0.25–0.81; *p* = 0.008). Age ≥50 years was not significantly linked to ypT0N0 (adjusted OR, 0.81; 95% CI, 0.46–1.41; *p* = 0.457).

### 3.8. Overall Survival

The median follow-up was 38.7 months (95% CI, 35.2–42.1 months). During follow-up, 26 deaths and 21 recurrences were reported (Table 12).

**Figure 3:**
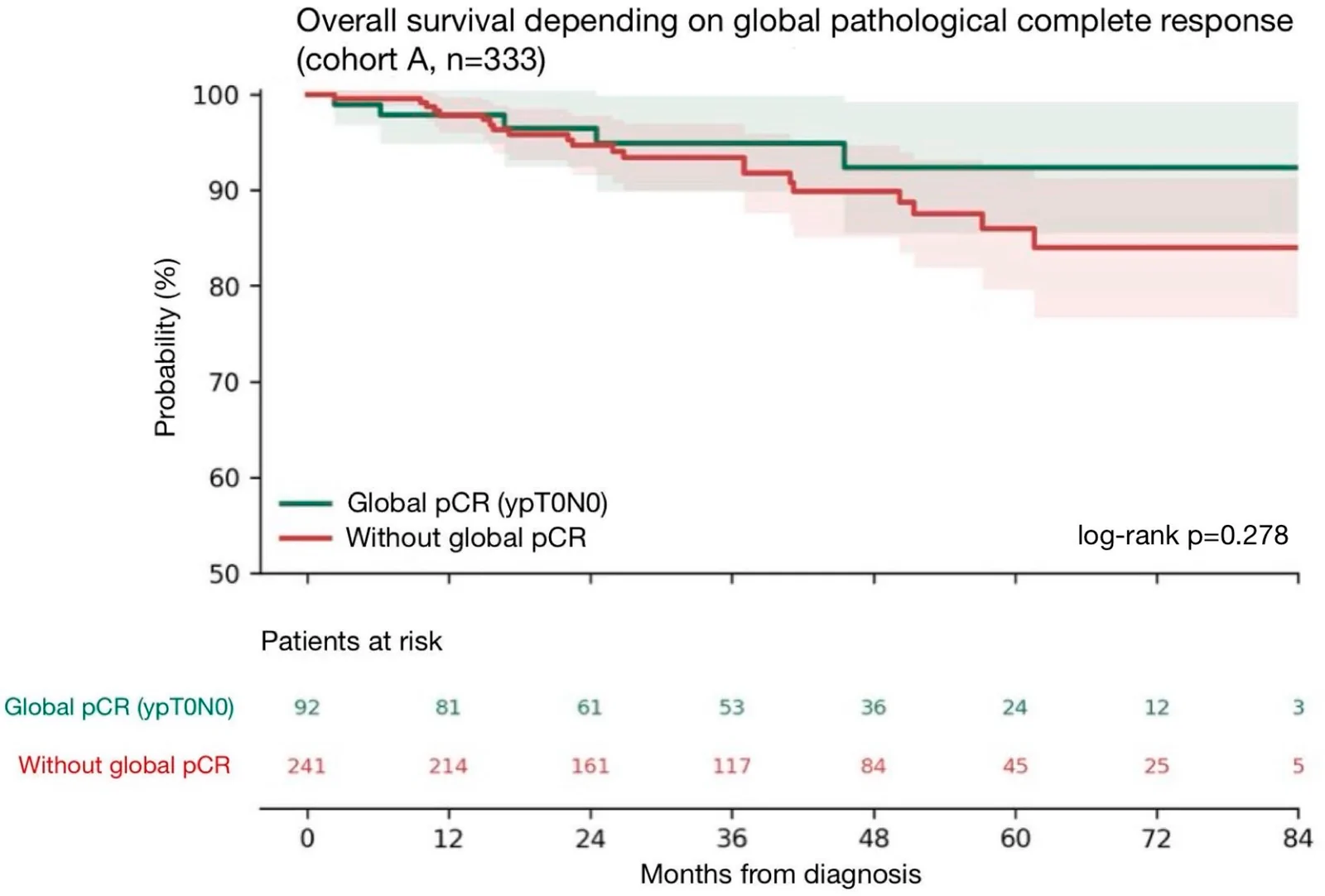
Overall survival depending on global pathological response (Cohort A). Numbers at risk are presented below each Kaplan–Meier curve to indicate the number of patients remaining under observation at the corresponding time points.

**Figure 4:**
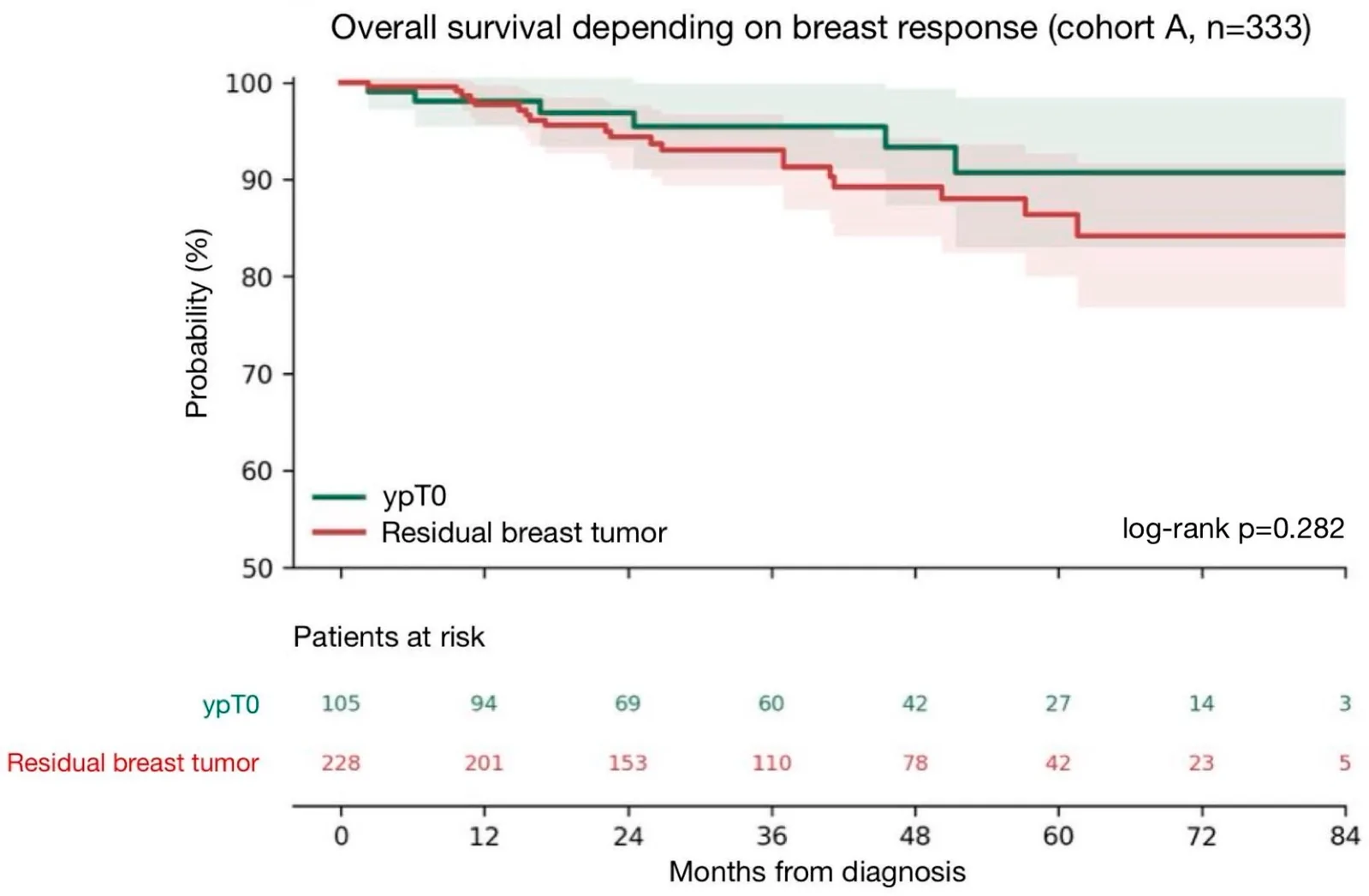
Overall survival depending on breast response (Cohort A). Numbers at risk are presented below each Kaplan–Meier curve to indicate the number of patients remaining under observation at the corresponding time points.

**Figure 5:**
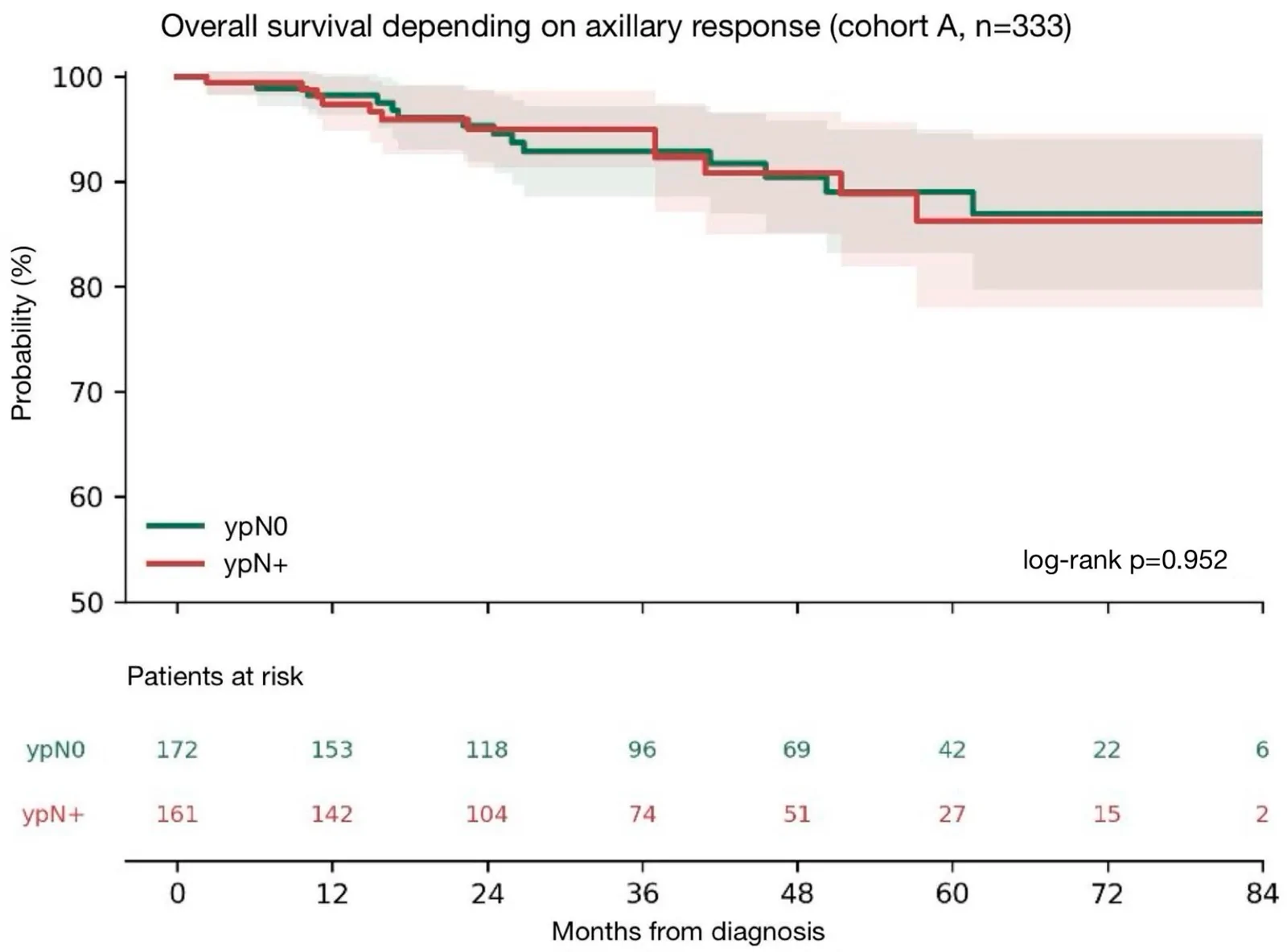
Overall survival depending on axillary response (Cohort A). Numbers at risk are presented below each Kaplan–Meier curve to indicate the number of patients remaining under observation at the corresponding time points.

Overall survival did not differ significantly according to overall pCR (ypT0N0; log-rank *p* = 0.278), axillary pathological complete response (ypN0; log-rank *p* = 0.952), or breast pathological complete response (ypT0; log-rank *p* = 0.282), as represented in Figure 3, Figure 4 and Figure 5. Three-year and five-year overall survival were 94.8% and 92.4%, respectively, among patients with ypT0N0, compared with 93.4% and 86.0% among patients without global pCR. Among patients with ypN0, 3-year and 5-year overall survival were 92.8% and 89.0%, compared with 95.0% and 86.3% among patients with ypN+. Among patients with ypT0, 3-year and 5-year overall survival were 95.5% and 90.7%, compared with 93.0% and 86.4% among patients with residual breast cancer.

Overall survival differed significantly according to molecular subtype (log-rank *p* = 0.032). Five-year overall survival was 74.9% for Luminal A-like, 91.4% for Luminal B-like, 93.1% for HER2-positive disease, and 81.0% for TNBC.

In univariate Cox regression analysis, overall pathological complete response (ypT0N0) had no significant relation with overall survival (HR, 0.59; 95% CI, 0.22–1.56; *p* = 0.284). Likewise, both ypN0 and ypT0 were not significantly associated with overall survival. Between the evaluated clinicopathological factors, the presence of ≥4 residual positive lymph nodes was significantly linked to worse overall survival (HR, 2.50; 95% CI, 1.09–5.76; *p* = 0.031). In multivariate analysis, ≥4 residual positive lymph nodes remained independently associated with worse overall survival (adjusted HR, 2.46; 95% CI, 1.01–5.98; *p* = 0.046), while ypT0N0 was not independently associated with overall survival (adjusted HR, 0.54; 95% CI, 0.18–1.61; *p* = 0.268). ER/PR positivity had no significant relation with overall survival (adjusted HR, 0.46; 95% CI, 0.20–1.10; *p* = 0.080).

### 3.9. Disease-Free Survival

**Figure 6:**
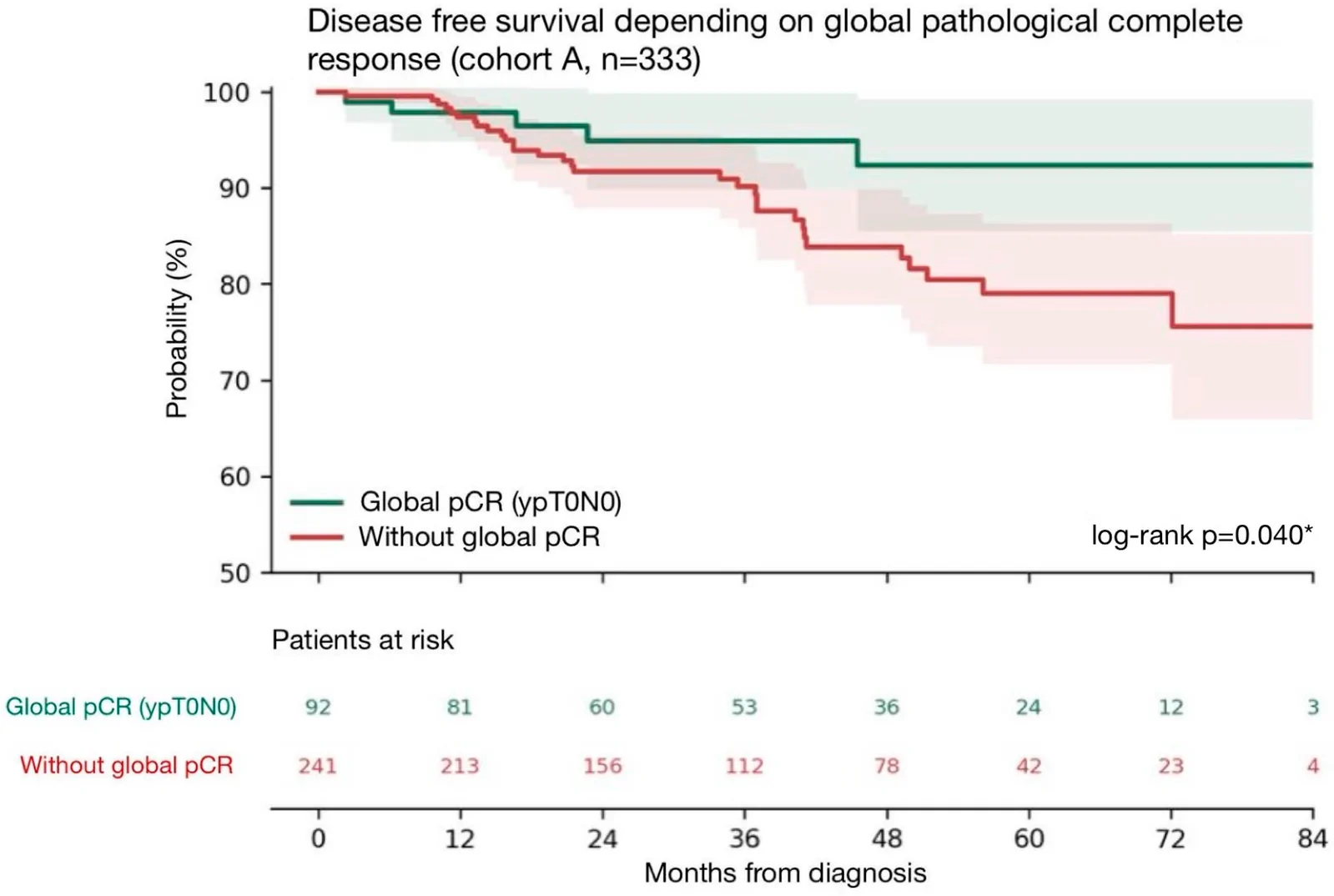
Disease-free survival depending on global pathological response (Cohort A). Numbers at risk are presented below each Kaplan–Meier curve to indicate the number of patients remaining under observation at the corresponding time points. * *p* < 0.05.

Disease-free survival differed significantly according to overall pCR (log-rank *p* = 0.040), as shown in Figure 6. Three-year and five-year disease-free survival were 94.8% and 92.4% among patients with ypT0N0, compared with 90.1% and 79.0% among patients without global pCR (Table 13).

**Figure 7:**
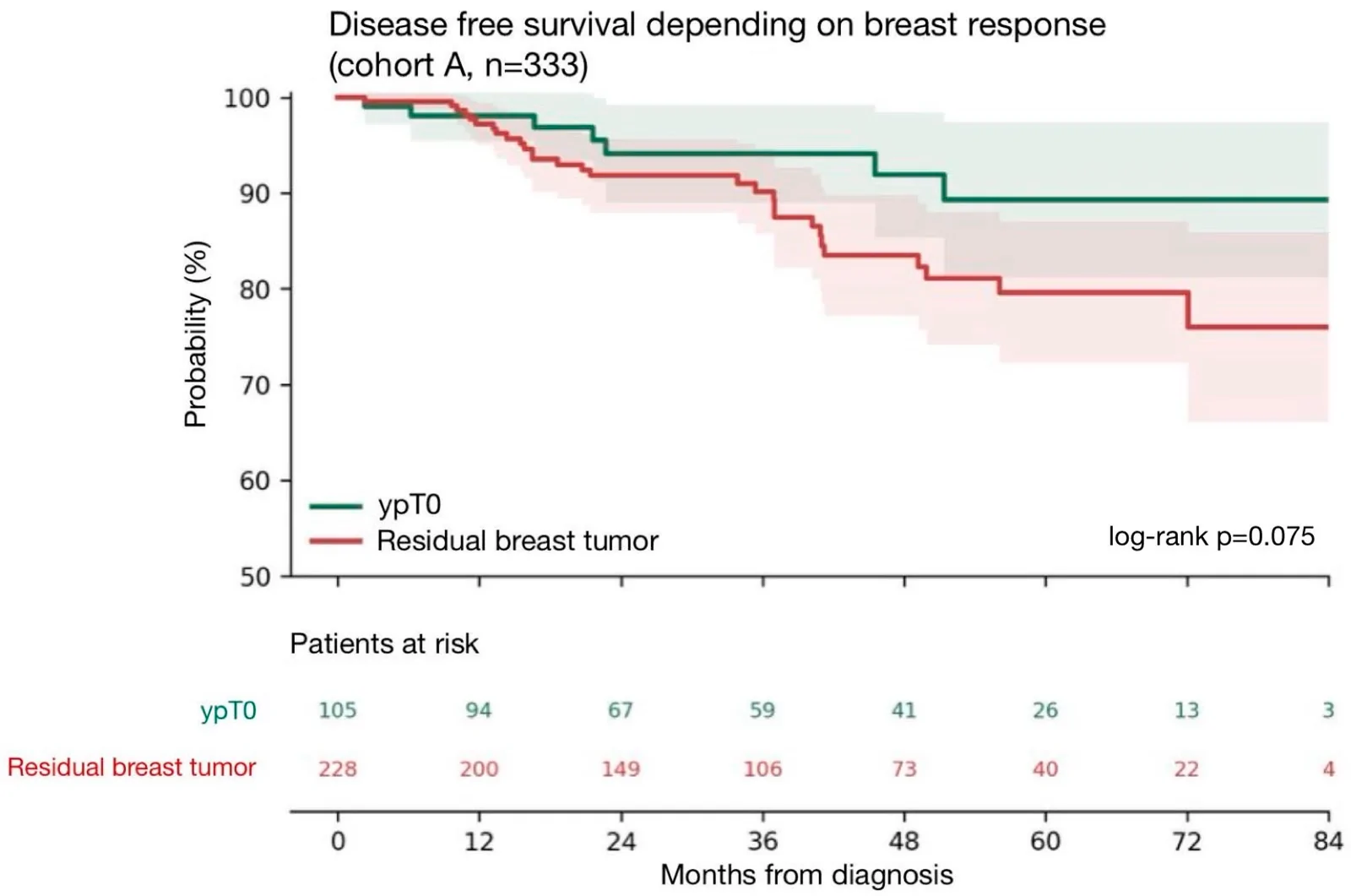
Disease-free survival depending on breast response (Cohort A). Numbers at risk are presented below each Kaplan–Meier curve to indicate the number of patients remaining under observation at the corresponding time points.

Breast pathological complete response was not significantly associated with disease-free survival (log-rank *p* = 0.075), as represented in Figure 7; three-year and five-year disease-free survival were 94.1% and 89.3% among patients with ypT0, compared with 90.2% and 79.6% among patients with residual breast tumor.

**Figure 8:**
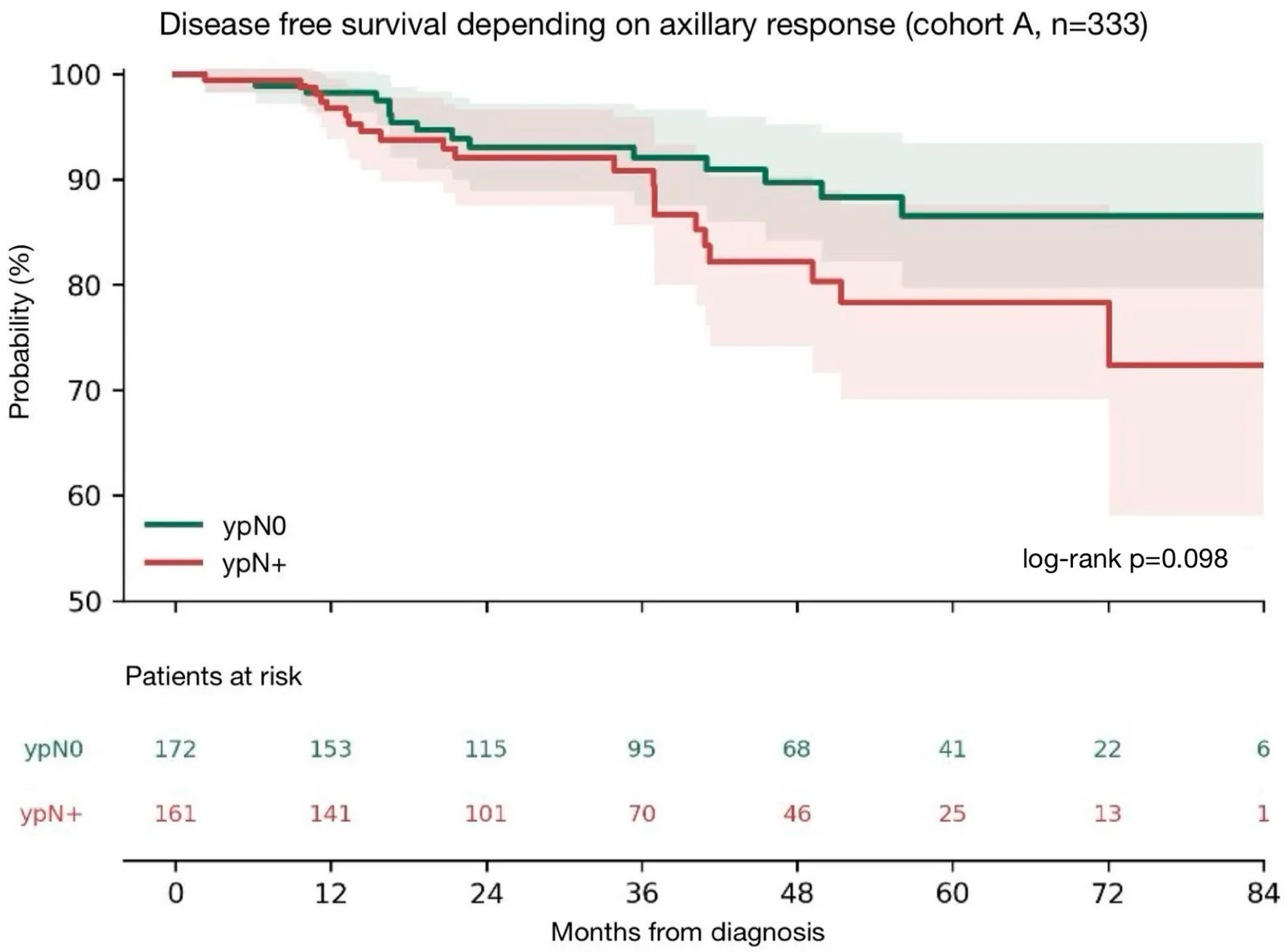
Disease-free survival depending on axillary response (Cohort A). Numbers at risk are presented below each Kaplan–Meier curve to indicate the number of patients remaining under observation at the corresponding time points.

Similarly, for axillary pathological response, the difference in disease-free survival did not reach statistical significance (log-rank *p* = 0.098), as represented in Figure 8. Three-year and five-year disease-free survival were 92.1% and 86.6% among patients with ypN0, compared with 90.8% and 78.4% among patients with ypN+.

In univariate Cox regression analysis, overall pCR (ypT0N0) had a significant association with improved disease-free survival (HR, 0.38; 95% CI, 0.15–0.99; *p* = 0.047), while ypN0 and ypT0 did not reach the level of statistical significance. The presence of ≥4 residual positive lymph nodes was significantly associated with worse disease-free survival (HR, 2.55; 95% CI, 1.26–5.20; *p* = 0.010). In multivariate analysis, ypT0N0 remained significantly associated with improved disease-free survival (adjusted HR, 0.35; 95% CI, 0.13–0.99; *p* = 0.048), while ≥4 residual positive lymph nodes remained independently linked to worse disease-free survival (adjusted HR, 2.27; 95% CI, 1.08–4.78; *p* = 0.031). ER/PR positivity had no significant association with disease-free survival (adjusted HR, 0.51; 95% CI, 0.24–1.07; *p* = 0.074) (Table 14 and Table 15).

## 4. Discussion

### 4.1. Interpretation of the Main Findings

A significant proportion of patients achieved axillary pCR following neoadjuvant systemic treatment. In the entire cohort, conventional ypN0 was observed in 50.4% of patients (282/560). An additional 16 patients had isolated tumor cells classified as ypN0(i+), resulting in 53.2% (298/560) when ypN0(i+) was included. Breast pathological complete response (ypT0) was observed in 31.2%. Overall pCR (ypT0N0) was achieved in 27.0%.

The axillary response was strongly dependent on tumor biology. HER2-positive and TNBC showed substantially higher ypN0 rates than the Luminal A-like and Luminal B-like subtypes, with ypN0 observed in 78.9% of HER2-positive tumors and 72.6% of TNBC, compared with 26.6% in Luminal A-like and 32.5% in Luminal B-like disease. The statistically significant association between molecular subtype and axillary response was maintained in multivariate analysis, in which HER2-positive disease remained independently associated with ypN0. Nottingham grade 3 was identified as an independent predictor of complete axillary response and lower TIL levels were associated with a diminished probability of ypN0. These findings indicate that axillary response was not uniform across the study population and was associated with biological and pathological tumor characteristics.

The relationship between breast and axillary pathological response stood out after the analysis. Patients achieving ypT0 had a higher probability of achieving ypN0, with a crude odds ratio (OR) of 13.09, decreasing to 4.75 after adjusting for molecular subtype, Nottingham grade, TILs and Ki-67. The treatment response in the breast did not always match the treatment response in the axilla: 12.4% of patients with breast pCR still had positive axillary disease, while 35.1% of patients with residual breast tumor had achieved ypN0. The sensitivity of ypT0 for predicting ypN0 was 53.5%, despite a high specificity of 91.9%. Therefore, based on the existing data, the pathological response of the breast and axilla should be considered related but non-interchangeable endpoints.

The association between breast and axillary response observed in the cohort supports the concept that breast pCR is linked to nodal clearance, particularly in HER2-positive and triple-negative disease. However, the sensitivity of 53.5% and negative predictive value of 64.9% highlight that breast pCR alone is not sufficient to reliably exclude residual axillary disease and therefore cannot replace pathological axillary assessment. Among patients who achieved breast pCR, ypN0 was observed in 95% of HER2-positive and 90% of triple-negative tumors, in comparison with 64% in Luminal B-like tumors. Even if these subtype-specific differences are clinically relevant and might be useful to identify patients with a higher probability of nodal clearance, they should be regarded as hypothesis-generating (given the size of the individual subgroups) and warrant validation in larger prospective cohort studies.

The survival analyses showed an exploratory association between overall pathological complete response and disease-free survival, with a significant log-rank *p* value of 0.040. On the other hand, the associations between axillary and breast response (ypN0 and ypT0) and disease-free survival did not reach conventional statistical significance. Overall survival differences according to global pCR, axillary pCR, and breast pCR were likewise not statistically significant in the reported analyses.

The survival analysis should be interpreted with caution and considered exploratory. The relatively small number of events (26 deaths for OS and 36 DFS events), together with a median follow-up of 38.7 months, limits the statistical power of the survival analyses and the robustness of multivariable Cox regression in this cohort. Hence, the absence of a statistically significant relation between ypN0 status and OS or DFS should not be regarded as evidence that axillary pathological response lacks prognostic relevance. Likewise, the association between global pCR and DFS should be interpreted cautiously, considering the borderline upper confidence limit of the adjusted hazard ratio (HR 0.35, 95% CI upper limit 0.99). These findings should be viewed as hypothesis-generating and require validation in larger cohorts with a greater number of clinical events and longer follow-up.

The relatively low 5-year OS which was observed in the Luminal A-like subgroup (74.9%), compared with 93.1% in the HER2-positive subgroup, should be interpreted with caution. This finding is not necessarily reflective of an intrinsic adverse prognosis of Luminal A-like disease and might have been caused by the limited number of events, differences in treatment characteristics, duration of follow-up and even the criteria used for molecular subtype classification. Thus, this observation should be considered hypothesis-generating rather than interpreted as evidence of a biological survival disadvantage in cases presenting Luminal A-like subtype.

Altogether, the results demonstrate a substantial pathological response in the axilla after neoadjuvant treatment, with marked heterogeneity according to tumor subtype Nottingham grade, TILs and Ki-67. The findings indicate that primary tumor response in the breast lacks sufficient sensitivity to accurately reflect axillary nodal clearance. While these insights inform our understanding of post-neoadjuvant nodal response, the present data do not directly justify or define a modified surgical approach to the axilla.

### 4.2. Overview of the Existing Evidence in the Literature

The available evidence in the literature supports that axillary response following neoadjuvant systemic therapy is strongly dependent on breast cancer subtype. In a systematic review and meta-analysis of initially clinically node-positive breast cancer, Samiei et al. mentioned the highest axillary pathological complete response rates in HER2-positive and TNBC, while lower rates were observed in HR-positive/HER2-negative disease [1]. These findings support that nodal response has a close relationship with tumor biology and that the likelihood of achieving ypN0 is not uniform between clinically node-positive patients [1].

In this context, De Rose et al. reported variability across axillary management strategies in clinical practice, as 60.4% of Italian radiation oncology centers performed ALND selectively and 37.6% routinely in patients with 1–2 positive sentinel lymph nodes, while the use of regional nodal irradiation following ALND omission also varied considerably [19]. Moreover, according to another study focusing on the pattern of practice in Italy, there exists an increasing trend toward ALND omission in selected cN+ patients achieving ypN0 status; however, this approach was adopted predominantly in cN1 disease, while management of cases with a higher nodal burden (like cN2 disease) remains less standardized [20].

An additional determinant of response is represented by the initial extent of nodal involvement. Myers et al. showed that higher clinical nodal stage was associated with a lower likelihood of achieving breast and axillary pathological complete response across several breast cancer molecular subtypes [2]. Hence, the favorable biological characteristics linked to nodal response do not fully overcome the impact of a higher baseline nodal burden [2].

Evidence addressing cN2/cN3 disease has only become available recently. In a large national cancer database, which included 20,402 patients diagnosed with cN2/cN3 breast cancer and treated with neoadjuvant chemotherapy, 36.9% achieved ypN0, while 63.1% presented residual nodal disease [3]. The probability of nodal clearance varied with respect to tumor characteristics, with ypN0 rates of 70% in HER2-positive tumors, 57% in TNBC, and 29% in hormone receptor-positive tumors [3]. This implies that complete nodal response is present in approximately one-third to two-fifths of patients with clinically advanced nodal disease, but that residual nodal disease remains, overall, more common [3].

Across the literature, the relationship between breast and axillary response is another discussed feature. Samiei et al. demonstrated a significant relation between pathological complete response in the breast and absence of axillary lymph node metastases following neoadjuvant systemic therapy in initially node-positive breast cancer patients [4]. The CALGB 40601 and 40603 analyses highlighted that breast response and pretreatment clinical nodal status are important predictors of ypN0 in HER2-positive and TNBC [5]. Ryu et al. emphasized that the concordance between breast and axillary pathological complete response decreased with increasing clinical nodal stage, emphasizing that breast response alone cannot consistently predict the final axillary pathological status in cases with greater baseline nodal burden [6].

The cN2-specific evidence further presents this limitation while demonstrating the strength of the association. In the study by Guo et al., which included 709 cases with cN2 HER2-positive or TNBC, 78.0% of patients who presented breast pCR also achieved axillary pCR [7]. Residual axillary nodal disease occurred in 77.4% of the patients who did not achieve breast pCR [7]. Thus, breast pCR identifies a subgroup with a higher probability of nodal clearance, but approximately one in five patients with breast pCR still presents residual axillary disease [7]. This finding is relevant when considering if breast response can be used as a basis for axillary surgical de-escalation strategies in cN2 disease.

Sheng et al. questioned whether breast pCR could be used to identify patients who may be candidates for omission of axillary surgery following neoadjuvant therapy [21]. In a multicenter retrospective cohort study on 864 patients, breast pCR was associated with an increased rate of axillary pCR (93.0% in patients with cN1 disease). In cases with cN2/3 disease achieving breast pCR, post-treatment imaging emphasized a false-negative rate of less than 5% for predicting nodal clearance. These findings suggested that selected patients with extensive baseline nodal disease and a favorable breast response might be considered for axillary surgical de-escalation [21]. However, considering that their study was designed retrospectively, these findings still require validation by prospective studies before omission of axillary staging can be routinely integrated into clinical practice.

Nazlı et al. explored the role of post-neoadjuvant axillary imaging for selecting patients with cN2 disease who can undergo axillary de-escalation [22]. In their retrospective cohort of 166 patients with biopsy-proven axillary metastases, radiologic nodal clearance on post-treatment ultrasound occurred in 36.7% of cases and was strongly associated with axillary pCR, which was present in 86.9% of patients with radiologically node-negative axillae in comparison with 5.7% of those with persistent radiologic nodal involvement (*p* < 0.001) [22]. Among patients with radiologic nodal clearance who underwent limited SLNB or targeted axilla dissection, 91.5% achieved ypN0(i−). This suggested that post-treatment ultrasound could provide useful additional information for selecting cN2 patients suitable for axillary de-escalation [22]. However, the authors emphasized that imaging should complement rather than replace surgical staging and multidisciplinary assessment.

Tinterri et al. evaluated the long-term outcomes of axillary de-escalation in breast cancer patients who were clinically node-positive and converted to ycN0 following neoadjuvant therapy [23]. In a cohort of 322 patients, those who underwent SLNB had significantly better recurrence-free, distant disease-free, overall and breast cancer-specific survival in comparison with patients in whom ALND was performed, while axillary recurrence rates remained low in both groups (0.6–2.1%) [23]. Even though these findings support the feasibility of axillary de-escalation in selected cases achieving clinical nodal response, the retrospective design and differences regarding baseline characteristics between treatment groups limit the ability to establish SLNB as equivalent to ALND.

Kim et al. conducted a study in which comparable long-term oncological outcomes between SLNB and ALND were reported for patients who achieved conversion (axillary ypN0) following neoadjuvant chemotherapy; ALND was associated with a higher rate of postoperative lymphedema [24]. Their findings further support the role of SLNB as an axillary staging approach with less morbidity, being performed in carefully selected cases presenting complete nodal response [24].

Further evidence supporting the ongoing shift toward axillary de-escalation derives from the OPBC-05/ICARO study, in which patients with residual isolated tumor cells (ypN0i+) after NAC were evaluated [25]. Although additional positive nodes were identified in 30% of patients who underwent ALND, only 5% presented macrometastatic disease, and axillary recurrence was rare among patients who were managed without ALND [25]. Therefore, the extent of residual nodal disease after the completion of NAC might be lower than expected in selected cases, even though their applicability to patients presenting with a higher initial nodal burden (such as cN2 disease) remains uncertain.

A review conducted by Ma et al. further emphasized that, even in selected patients with cN2 disease (particularly those with HER2-positive or triple-negative breast cancer who achieve a favorable pathological response), might represent candidates for axillary de-escalation [26]. This is attributed to the fact that evidence deriving from primary studies on the omission of ALND is limited, and thus ALND remains the standard strategy for management in these settings [26].

The prognostic implications of axillary response have also been discussed. In the GeparOcto trial, pathological response in the breast and axillary lymph nodes following neoadjuvant systemic treatment was related to disease-free survival in cases with initially node-positive breast cancer [8]. Jankowski et al. reported an association between axillary pathological response, breast cancer subtype, and survival outcomes [9]. These findings highlight that nodal response is not only a marker of treatment response, but it also has prognostic relevance. The prognostic value of ypN0 must be interpreted in the context of the original disease burden. Wang et al. reported that patients who converted from node-positive to ypN0 following neoadjuvant chemotherapy had less favorable 10-year outcomes in comparison with patients who were node-negative at baseline, indicating that pathological nodal clearance does not diminish the prognostic impact of initially node-positive disease [10].

From a surgical point of view, these findings have relevance for cN2 disease. The high rates of nodal response which were observed in HER2-positive and triple-negative tumors, together with the strong relationship between breast pCR and ypN0, provide a rationale for investigating if axillary surgery can be safely de-escalated in selected patients [1,7]. The recent cN2-specific study conducted by Guo et al. addressed this question and indicated that patients with cN2 HER2-positive or triple-negative breast cancer who achieve breast pCR might constitute a category in whom less extensive axillary surgery could be considered [7]. However, this evidence is observational and retrospective.

A major limitation of the existing literature is represented by the relative scarcity of cN2-specific evidence in comparison with the broader clinically node-positive population. Some of the evidence regarding breast–axilla concordance and axillary pCR originates from mixed cN+ cohorts, while the more recent studies which focused on cN2/cN3 disease have retrospective designs [1,3,4,5,6,7]. Moreover, the available evidence indicates an association between nodal clearance and improved outcomes but does not establish that conversion to ypN0 has the same prognosis as being node-negative at diagnosis [8,9,10].

Finally, direct comparison across studies is restricted by heterogeneity in terms of clinical nodal stage, tumor subtype, neoadjuvant regimens, definitions of pathological response and study populations.

### 4.3. Strengths and Limitations of the Current Study

A major strength of the study lies in its robust sample size (n = 560), together with the availability of detailed clinicopathological variables and distinct pathological response metrics. By evaluating breast and axillary responses independently rather than relying solely on overall pCR (pCR), their precise interplay was included and characterized. In addition, the study evaluated multiple potential predicting factors using both univariate and multivariate logistic regression and incorporated survival outcomes with a reported median follow-up of 38.7 months, which adds significant clinical depth.

The study provides a detailed assessment of the association between molecular subtype and pathological response, as well as measures of concordance and predictive performance between breast and axillary clearance. Adjusting the relationship between ypT0 and ypN0 for molecular subtype, Nottingham grade, TILs, and Ki-67, substantially enhances the validity of these findings.

Apart from the retrospective design and inconsistent availability of certain clinical and treatment-related variables, multiple additional limitations should be acknowledged and taken into consideration while interpreting the findings.

First, the current study was conducted at a single tertiary oncology institution. Even if the cohort was relatively large, the institutional setting and referral patterns might limit the generalizability of the results to other centers, (particularly centers with different patient populations, treatment protocols, diagnostic pathways, or surgical practices). The observed pathological response rates might reflect, at least partially, the characteristics of patients referred to our specialized oncology center.

Second, heterogeneity regarding the neoadjuvant systemic treatment represents an important limitation. Most of the patients underwent neoadjuvant chemotherapy, while a smaller proportion received endocrine therapy. The available dataset does not provide a sufficiently detailed treatment-level characterization in order to allow robust comparisons between specific systemic treatment regimens. For instance, treatment components that might influence pathological response with respect to molecular subtype cannot be assessed in a standardized manner. Therefore, the observed relations between tumor biology and pathological response should not be interpreted independently of treatment heterogeneity. In addition, the study period (2017–2025) encompassed changes in neoadjuvant treatment recommendations, with increasing use of dual HER2 blockade in HER2-positive disease, the incorporation of platinum-based chemotherapy, and immunotherapy in selected cases of triple-negative breast cancer. Treatment was therefore adapted in accordance with contemporary guidelines and institutional protocols applicable at the time of diagnosis. As the retrospective database did not systematically capture the specific systemic regimens and targeted or immunotherapeutic agents administered in each case, the observed differences in nodal response should be viewed with caution (particularly when comparing molecular subtypes across the entire study period).

Third, both the definition and documentation of cN2 disease were based on clinical and imaging assessments, combined with cytological or histological confirmation of nodal involvement performed in accordance with institutional practice. This may introduce some degree of heterogeneity in baseline nodal assessment. In addition, the study population was restricted to cases who proceeded to surgery with axillary evaluation following completion of neoadjuvant treatment. Patients who had incomplete or abandoned neoadjuvant treatment, lacked surgical axillary evaluation, or those who had incomplete key pathological information were excluded. This might have introduced selection bias. Another limitation is that the institutional database did not divide cN2 cases into cN2a and cN2b disease. Therefore, the present analysis is not able to provide a reliable estimate regarding the proportion of these two clinical presentations.

Fourth, an additional limitation is that post-neoadjuvant systemic treatment was not systematically collected in the present analysis. Subsequent treatment might have differed according to residual disease, molecular subtype, and evolving treatment standards during the study period and may have materially influenced DFS and OS. Therefore, the observed associations between pathological response and survival cannot be interpreted independently of potential differences in post-neoadjuvant management.

Fifth, another limitation concerns the distinction between the total cohort and Cohort A. Out of the 560 patients included in the overall pathological analysis, only 333 completed both neoadjuvant and adjuvant treatment at the Institute of Oncology “Prof. Dr. Ion Chiricuță” Cluj-Napoca. Although the 227 patients who received adjuvant treatment outside our institution continued regular clinical follow-up, detailed information regarding their subsequent treatment and long-term management was not systematically available within the institutional database. This fact is particularly relevant for survival analyses, which were restricted to patients in Cohort A meeting the predefined follow-up criteria. The survival results should not be directly extrapolated to the entire cohort of 560 patients. Differences in treatment or follow-up outside the institution might have been responsible for long-term outcomes.

Sixth, the multivariable analyses should be interpreted with caution. For some adjusted estimates, the associated confidence intervals were very wide (particularly for HER2-positive disease and TNBC), indicating possible sparse-data effects. Furthermore, the limited number of events within some biological subgroups might have decreased model stability and increased susceptibility to potential confounding. The magnitude of these adjusted odds ratios should, therefore, not be interpreted as precise estimates of treatment-independent effect. These analyses should be regarded as exploratory and hypothesis-generating. In the ypT0N0 model, subtype effects could not be estimated because no Luminal A-like tumor achieved ypT0N0.

Seventh, the relatively limited number of deaths and recurrences is another important fact. Only 26 deaths and 21 recurrences were reported during the follow-up period, and the median follow-up was equal to 38.7 months. This limits the statistical power of survival analyses and increases uncertainty in estimating long-term outcomes. Non-significant associations between pathological response and survival should be interpreted with caution.

In the end, even if multivariate models were applied to adjust for several clinicopathological variables, residual confounding cannot be excluded. As in any retrospective observational study, potentially relevant factors could have been incompletely recorded or unavailable for analysis. Hence, the associations which were identified between molecular subtype, histological grade, TILs, Ki-67 and pathological response should not be regarded as causal relationships. Prospective, multicenter studies, with standardized neoadjuvant treatment protocols, uniform axillary staging, standardized pathological assessment and longer follow-up periods are required to confirm these findings and to establish whether pathological response can safely lead to individualized axillary surgical strategies.

## 5. Conclusions

In this retrospective cohort study on patients diagnosed with cN2 breast cancer, axillary pCR following neoadjuvant systemic treatment occurred in approximately half of the patients. Axillary response presented heterogeneity with respect to tumor biology. Particularly high ypN0 rates were observed in HER2-positive and triple-negative disease. HER2-positive status and Nottingham grade 3 were associated with a greater likelihood of achieving complete axillary response, whereas low TIL levels were linked to a lower probability of achieving nodal clearance.

Breast and axillary pathological responses were significantly linked, but the degree of concordance was insufficient to consider them interchangeable endpoints. A relevant proportion of patients had residual axillary disease in spite of the complete breast response, whereas multiple patients with residual breast tumor obtained complete axillary response. These findings highlight that assessment of the primary tumor alone cannot reliably determine the pathological status of the axilla following completion of neoadjuvant treatment.

Overall pCR was related to improved DFS, while isolated breast or axillary pathological complete response did not show statistically significant associations with survival in our cohort. The absence of statistically significant associations for several survival endpoints should be regarded in the context of the relatively short median follow-up and the limited number of events.

Our findings support the concept that post-neoadjuvant axillary response is dependent on tumor biology and should be viewed as an independent clinical and pathological endpoint. However, the present study does not bring sufficient evidence in order to recommend omission or even modification of axillary surgery in patients with cN2 disease. To determine whether selected patients with a favorable biological profile and documented nodal response might safely undergo less extensive axillary surgery, larger multicenter studies with standardized systemic treatment, axillary assessment and longer follow-up are required.

## Figures and Tables

**Table 1:** Baseline clinicopathological characteristics of the study population.

Variable	Total (n = 560)	Cohort A (n = 333)
Age at diagnosis (years), mean	56.2	56.0
Menstrual status		
Premenopausal/cyclic	179 (32.0%)	102 (30.6%)
Postmenopausal	381 (68.0%)	231 (69.4%)
Clinical stage		
IIB	2 (0.4%)	0 (0.0%)
III	1 (0.2%)	0 (0.0%)
IIIA	547 (97.7%)	329 (98.8%)
IIIB	10 (1.8%)	4 (1.2%)
cT category		
cT1–2	398 (71.1%)	242 (72.7%)
cT3–4	162 (28.9%)	91 (27.3%)
Molecular subtype		
Luminal A-like	109 (19.5%)	57 (17.1%)
Luminal B-like	206 (36.8%)	123 (36.9%)
HER2-positive ^a^	128 (22.9%)	91 (27.3%)
Triple-negative breast cancer (TNBC)	117 (20.9%)	62 (18.6%)
ER/PR status		
Positive	386 (68.9%)	229 (68.8%)
Negative	174 (31.1%)	104 (31.2%)
Histological subtype		
Invasive ductal carcinoma	516 (92.1%)	315 (94.6%)
Invasive lobular carcinoma	27 (4.8%)	11 (3.3%)
Mucinous carcinoma	15 (2.7%)	6 (1.8%)
Apocrine carcinoma	1 (0.2%)	0 (0.0%)
Cribriform carcinoma	1 (0.2%)	1 (0.3%)
Nottingham grade		
G1	84 (15.0%)	41 (12.3%)
G2	253 (45.2%)	158 (47.4%)
G3	223 (39.8%)	134 (40.2%)
Ki-67 (%), median (IQR)	25.0 (15.0–50.0)	30.0 (15.0–50.0)
Ki-67 <20%		
Yes	175 (31.2%)	102 (30.6%)
No	385 (68.8%)	231 (69.4%)
TILs (%), median (IQR)	10.0 (5.0–30.0)	10.0 (5.0–30.0)
TILs <10%		
Yes	236 (42.1%)	147 (44.1%)
No	324 (57.9%)	186 (55.9%)
Associated DCIS (%), median (IQR)	1.0 (0.0–5.0)	1.0 (0.0–5.0)
DCIS <10%		
Yes	439 (78.4%)	262 (78.7%)
No	121 (21.6%)	71 (21.3%)
Type of neoadjuvant treatment		
Chemotherapy (CT)	506 (90.4%)	299 (89.8%)
Endocrine therapy (ET)	54 (9.6%)	34 (10.2%)
Type of breast surgery		
Mastectomy	409 (73.0%)	236 (70.9%)
Breast-conserving surgery	151 (27.0%)	97 (29.1%)
Resection margins		
R0	555 (99.1%)	329 (98.8%)
R1	5 (0.9%)	4 (1.2%)
Number of excised lymph nodes, median (IQR)	15.0 (12.0–19.0)	15.0 (12.0–19.0)
Number of positive lymph nodes, median (IQR)	0.0 (0.0–3.0)	0.0 (0.0–2.0)

Abbreviations: IQR, interquartile range; ER, estrogen receptor; PR, progesterone receptor; HER2, human epidermal growth factor receptor 2; TNBC, triple-negative breast cancer; TILs, tumor-infiltrating lymphocytes; DCIS, ductal carcinoma in situ; CT, chemotherapy; ET, endocrine therapy. ^a^ HER2-positive status was assessed independently of molecular subtype classification. Of the 130 HER2-positive tumors, 128 were classified as the HER2-positive molecular subtype, while two hormone receptor-positive/HER2-positive tumors were classified as Luminal B-like according to the St. Gallen criteria [16].

**Table 2:** Pathological response rates.

Response	Total, n/N	% (95% CI)	Cohort A, n/N (%)
Breast pathological complete response (ypT0)	175/560	31.2% (27.5–35.2)	105/333 (31.5%)
Axillary pathological complete response (ypN0)	282/560	50.4% (46.2–54.5)	172/333 (51.7%)
Overall pCR (ypT0N0)	151/560	27.0% (23.4–30.8)	92/333 (27.6%)
ypN0, including ypN0(i+)	298/560	53.2% (49.1–57.3)	184/333 (55.3%)

Abbreviations: CI, confidence interval; ypT0, breast pathological complete response; ypN0, axillary pathological complete response; ypT0N0, overall pathological complete response.

**Table 3:** Pathological response rates according to molecular subtype.

Molecular Subtype	n	ypT0, n (%)	ypN0, n (%)	ypT0N0, n (%)
Luminal A-like	109	2 (1.8%)	29 (26.6%)	0 (0.0%)
Luminal B-like	206	26 (12.6%)	67 (32.5%)	17 (8.3%)
HER2-positive	128	85 (66.4%)	101 (78.9%)	80 (62.5%)
TNBC	117	62 (53.0%)	85 (72.6%)	54 (46.2%)
Total	560	175 (31.2%)	282 (50.4%)	151 (27.0%)
*p*-value (χ^2^ test)		<0.001	<0.001	<0.001

Abbreviations: ypT0, breast pathological complete response; ypN0, axillary pathological complete response; ypT0N0, overall pathological complete response; HER2, human epidermal growth factor receptor 2; TNBC, triple-negative breast cancer.

**Table 4:** Pathological response rates according to clinical T category.

Clinical T Category	n	ypT0, n (%)	ypN0, n (%)	ypT0N0, n (%)
cT1–2	398	132 (33.2%)	200 (50.3%)	114 (28.6%)
cT3–4	162	43 (26.5%)	82 (50.6%)	37 (22.8%)
Total	560	175 (31.2%)	282 (50.4%)	151 (27.0%)
*p*-value (χ^2^ test)		0.152	1.000	0.194

Abbreviations: ypT0, breast pathological complete response; ypN0, axillary pathological complete response; ypT0N0, overall pathological complete response.

**Table 5:** Univariable predictors of breast pathological complete response (ypT0).

Variable	ypT0 in Index Category, n/N (%)	ypT0 in Reference Category, n/N (%)	Crude OR (95% CI)	*p*-Value
Age ≥50 years (vs. <50 years), Cohort A	62/227 (27.3%)	43/106 (40.6%)	0.55 (0.34–0.89)	0.022
Age ≥50 years (vs. <50 years), entire cohort	106/374 (28.3%)	69/186 (37.1%)	0.67 (0.46–0.97)	0.045
Postmenopausal (vs. premenopausal), Cohort A	62/231 (26.8%)	43/102 (42.2%)	0.50 (0.31–0.82)	0.008
Postmenopausal (vs. premenopausal), entire cohort	105/381 (27.6%)	70/179 (39.1%)	0.59 (0.41–0.86)	0.008
Nottingham grade 3 (vs. G1–2), Cohort A	81/134 (60.4%)	24/199 (12.1%)	11.14 (6.43–19.31)	<0.001
Nottingham grade 3 (vs. G1–2), entire cohort	140/223 (62.8%)	35/337 (10.4%)	14.55 (9.35–22.67)	<0.001
Ki-67 <20% (vs. ≥20%), Cohort A	5/102 (4.9%)	100/231 (43.3%)	0.07 (0.03–0.17)	<0.001
Ki-67 <20% (vs. ≥20%), entire cohort	7/175 (4.0%)	168/385 (43.6%)	0.05 (0.02–0.12)	<0.001
TILs <10% (vs. ≥10%), Cohort A	16/147 (10.9%)	89/186 (47.8%)	0.13 (0.07–0.24)	<0.001
TILs <10% (vs. ≥10%), entire cohort	21/236 (8.9%)	154/324 (47.5%)	0.11 (0.07–0.18)	<0.001
DCIS <10% (vs. ≥10%), Cohort A	89/262 (34.0%)	16/71 (22.5%)	1.77 (0.96–3.26)	0.090
DCIS <10% (vs. ≥10%), entire cohort	149/439 (33.9%)	26/121 (21.5%)	1.88 (1.17–3.02)	0.012
ER/PR positive (vs. negative), Cohort A	42/229 (18.3%)	63/104 (60.6%)	0.15 (0.09–0.24)	<0.001
ER/PR positive (vs. negative), entire cohort	65/386 (16.8%)	110/174 (63.2%)	0.12 (0.08–0.18)	<0.001
HER2-positive (vs. negative), Cohort A	60/92 (65.2%)	45/241 (18.7%)	8.17 (4.77–13.98)	<0.001
HER2-positive (vs. negative), entire cohort	85/130 (65.4%)	90/430 (20.9%)	7.14 (4.64–10.97)	<0.001
Invasive ductal carcinoma (vs. non-ductal), Cohort A	105/315 (33.3%)	0/18 (0.0%)	Not estimable	0.007
Invasive ductal carcinoma (vs. non-ductal), entire cohort	171/516 (33.1%)	4/44 (9.1%)	4.96 (1.74–14.08)	0.002
Neoadjuvant chemotherapy (vs. endocrine therapy), Cohort A	104/299 (34.8%)	1/34 (2.9%)	17.60 (2.37–130.52)	<0.001
Neoadjuvant chemotherapy (vs. endocrine therapy), entire cohort	173/506 (34.2%)	2/54 (3.7%)	13.51 (3.25–56.12)	<0.001
cT3–4 (vs. cT1–2), Cohort A	22/91 (24.2%)	83/242 (34.3%)	0.61 (0.35–1.06)	0.101
cT3–4 (vs. cT1–2), entire cohort	43/162 (26.5%)	132/398 (33.2%)	0.73 (0.48–1.09)	0.152
Molecular subtype, Cohort A				<0.001
Luminal A-like	2/57 (3.5%)		1.00	
Luminal B-like	14/123 (11.4%)		3.53 (0.78–16.10)	0.103
HER2-positive	60/91 (65.9%)		53.23 (12.16–232.89)	<0.001
TNBC	29/62 (46.8%)		24.17 (5.41–107.93)	<0.001
Molecular subtype, entire cohort				<0.001
Luminal A-like	2/109 (1.8%)		1.00	
Luminal B-like	26/206 (12.6%)		7.73 (1.80–33.21)	0.006
HER2-positive	85/128 (66.4%)		105.76 (24.90–449.10)	<0.001
TNBC	62/117 (53.0%)		60.31 (14.22–255.87)	<0.001

Abbreviations: OR, odds ratio; CI, confidence interval; ypT0, breast pathological complete response; TILs, tumor-infiltrating lymphocytes; DCIS, ductal carcinoma in situ; ER, estrogen receptor; PR, progesterone receptor; HER2, human epidermal growth factor receptor 2; TNBC, triple-negative breast cancer.

**Table 6:** Multivariable predictors of breast pathological complete response (ypT0).

Variable	Adjusted OR (95% CI)	*p*-Value
Age ≥50 years, Cohort A	0.72 (0.37–1.37)	0.316
Age ≥50 years, entire cohort	0.84 (0.50–1.40)	0.495
Nottingham grade 3, Cohort A	3.21 (1.53–6.76)	0.002
Nottingham grade 3, entire cohort	3.68 (2.03–6.64)	<0.001
Ki-67 <20%, Cohort A	0.19 (0.06–0.61)	0.005
Ki-67 <20%, entire cohort	0.22 (0.09–0.56)	0.001
TILs <10%, Cohort A	0.43 (0.21–0.91)	0.027
TILs <10%, entire cohort	0.31 (0.17–0.57)	<0.001
DCIS <10%, Cohort A	2.27 (1.02–5.07)	0.045
DCIS <10%, entire cohort	1.92 (1.03–3.59)	0.041
Luminal B-like subtype (vs. Luminal A-like), Cohort A	0.90 (0.17–4.79)	0.899
Luminal B-like subtype (vs. Luminal A-like), entire cohort	2.58 (0.55–12.03)	0.228
HER2-positive subtype (vs. Luminal A-like), Cohort A	7.53 (1.45–39.23)	0.017
HER2-positive subtype (vs. Luminal A-like), entire cohort	18.59 (4.00–86.48)	<0.001
TNBC subtype (vs. Luminal A-like), Cohort A	1.88 (0.33–10.78)	0.481
TNBC subtype (vs. Luminal A-like), entire cohort	5.73 (1.18–27.83)	0.030
cT3–4 (vs. cT1–2), Cohort A	0.37 (0.18–0.76)	0.006
cT3–4 (vs. cT1–2), entire cohort	0.46 (0.27–0.80)	0.005

Abbreviations: OR, odds ratio; CI, confidence interval; TILs, tumor-infiltrating lymphocytes; DCIS, ductal carcinoma in situ; HER2, human epidermal growth factor receptor 2; TNBC, triple-negative breast cancer.

**Table 7:** Univariable predictors of axillary pathological complete response (ypN0).

Variable	ypN0 in Index Category, n/N (%)	ypN0 in Reference Category, n/N (%)	Crude OR (95% CI)	*p*-Value
Age ≥50 years (vs. <50 years), Cohort A	107/227 (47.1%)	65/106 (61.3%)	0.56 (0.35–0.90)	0.022
Age ≥50 years (vs. <50 years), entire cohort	181/374 (48.4%)	101/186 (54.3%)	0.79 (0.55–1.12)	0.220
Postmenopausal (vs. premenopausal), Cohort A	108/231 (46.8%)	64/102 (62.7%)	0.52 (0.32–0.84)	0.010
Postmenopausal (vs. premenopausal), entire cohort	181/381 (47.5%)	101/179 (56.4%)	0.70 (0.49–1.00)	0.060
Nottingham grade 3 (vs. G1–2), Cohort A	104/134 (77.6%)	68/199 (34.2%)	6.68 (4.05–11.02)	<0.001
Nottingham grade 3 (vs. G1–2), entire cohort	173/223 (77.6%)	109/337 (32.3%)	7.24 (4.91–10.68)	<0.001
Ki-67 <20% (vs. ≥20%), Cohort A	30/102 (29.4%)	142/231 (61.5%)	0.26 (0.16–0.43)	<0.001
Ki-67 <20% (vs. ≥20%), entire cohort	48/175 (27.4%)	234/385 (60.8%)	0.24 (0.17–0.36)	<0.001
TILs <10% (vs. ≥10%), Cohort A	37/147 (25.2%)	135/186 (72.6%)	0.13 (0.08–0.21)	<0.001
TILs <10% (vs. ≥10%), entire cohort	48/236 (20.3%)	234/324 (72.2%)	0.10 (0.07–0.15)	<0.001
DCIS <10% (vs. ≥10%), Cohort A	144/262 (55.0%)	28/71 (39.4%)	1.87 (1.10–3.20)	0.029
DCIS <10% (vs. ≥10%), entire cohort	233/439 (53.1%)	49/121 (40.5%)	1.66 (1.10–2.50)	0.019
ER/PR positive (vs. negative), Cohort A	90/229 (39.3%)	82/104 (78.8%)	0.17 (0.10–0.30)	<0.001
ER/PR positive (vs. negative), entire cohort	147/386 (38.1%)	135/174 (77.6%)	0.18 (0.12–0.27)	<0.001
HER2-positive (vs. negative), Cohort A	74/92 (80.4%)	98/241 (40.7%)	6.00 (3.37–10.67)	<0.001
HER2-positive (vs. negative), entire cohort ^a^	101/130 (77.7%)	181/430 (42.1%)	4.79 (3.04–7.55)	<0.001
Invasive ductal carcinoma (vs. non-ductal), Cohort A	163/315 (51.7%)	9/18 (50.0%)	1.07 (0.41–2.77)	1.000
Invasive ductal carcinoma (vs. non-ductal), entire cohort	265/516 (51.4%)	17/44 (38.6%)	1.68 (0.89–3.15)	0.144
Neoadjuvant chemotherapy (vs. endocrine therapy), Cohort A	160/299 (53.5%)	12/34 (35.3%)	2.11 (1.01–4.42)	0.067
Neoadjuvant chemotherapy (vs. endocrine therapy), entire cohort	264/506 (52.2%)	18/54 (33.3%)	2.18 (1.21–3.94)	0.013
cT3–4 (vs. cT1–2), Cohort A	50/91 (54.9%)	122/242 (50.4%)	1.20 (0.74–1.95)	0.539
cT3–4 (vs. cT1–2), entire cohort	82/162 (50.6%)	200/398 (50.3%)	1.01 (0.70–1.46)	1.000
Molecular subtype, Cohort A				<0.001
Luminal A-like	13/57 (22.8%)		1.00	
Luminal B-like	40/123 (32.5%)		1.63 (0.79–3.37)	0.186
HER2-positive	74/91 (81.3%)		14.73 (6.54–33.21)	<0.001
TNBC	45/62 (72.6%)		8.96 (3.89–20.61)	<0.001
Molecular subtype, entire cohort				<0.001
Luminal A-like	29/109 (26.6%)		1.00 (reference)	
Luminal B-like	67/206 (32.5%)		1.33 (0.79–2.23)	0.278
HER2-positive	101/128 (78.9%)		10.32 (5.66–18.82)	<0.001
TNBC	85/117 (72.6%)		7.33 (4.07–13.19)	<0.001

Abbreviations: OR, odds ratio; CI, confidence interval; ypN0, axillary pathological complete response; TILs, tumor-infiltrating lymphocytes; DCIS, ductal carcinoma in situ; ER, estrogen receptor; PR, progesterone receptor; HER2, human epidermal growth factor receptor 2; TNBC, triple-negative breast cancer. ^a^ HER2-positive status was assessed independently of molecular subtype classification; therefore, the denominator of 130 includes all HER2-positive tumors in the entire cohort, including two hormone receptor-positive/HER2-positive tumors classified as Luminal B-like.

**Table 8:** Multivariable predictors of axillary pathological complete response (ypN0).

Variable	Adjusted OR (95% CI)	*p*-Value
Age ≥50 years, Cohort A	0.74 (0.42–1.33)	0.316
Age ≥50 years, entire cohort	1.04 (0.67–1.63)	0.858
Nottingham grade 3, Cohort A	2.04 (1.02–4.07)	0.043
Nottingham grade 3, entire cohort	2.27 (1.31–3.92)	0.003
Ki-67 <20%, Cohort A	0.79 (0.38–1.62)	0.514
Ki-67 <20%, entire cohort	0.63 (0.36–1.09)	0.102
TILs <10%, Cohort A	0.26 (0.15–0.46)	<0.001
TILs <10%, entire cohort	0.18 (0.11–0.27)	<0.001
DCIS <10%, Cohort A	1.98 (1.02–3.84)	0.043
DCIS <10%, entire cohort	1.60 (0.97–2.65)	0.066
Luminal B-like subtype (vs. Luminal A-like), Cohort A	1.14 (0.48–2.68)	0.772
Luminal B-like subtype (vs. Luminal A-like), entire cohort	0.82 (0.43–1.55)	0.535
HER2-positive subtype (vs. Luminal A-like), Cohort A	5.14 (1.91–13.85)	0.001
HER2-positive subtype (vs. Luminal A-like), entire cohort	3.05 (1.44–6.49)	0.004
TNBC subtype (vs. Luminal A-like), Cohort A	2.47 (0.82–7.41)	0.106
TNBC subtype (vs. Luminal A-like), entire cohort	1.52 (0.67–3.45)	0.316
cT3–4 (vs. cT1–2), Cohort A	1.11 (0.61–2.02)	0.744
cT3–4 (vs. cT1–2), entire cohort	0.85 (0.54–1.35)	0.487

Abbreviations: OR, odds ratio; CI, confidence interval; TILs, tumor-infiltrating lymphocytes; DCIS, ductal carcinoma in situ; HER2, human epidermal growth factor receptor 2; TNBC, triple-negative breast cancer.

**Table 9:** Association between breast and axillary pathological response.

Indicator	Value	Notes
Residual breast tumor + ypN+	148	44.4%
Residual breast tumor + ypN0	80	24.0%
ypT0 + ypN+	13	3.9%
ypT0 + ypN0	92	27.6%
Crude OR (ypT0 → ypN0)	13.09	95% CI, 6.89–24.86; *p* < 0.001
Adjusted OR (subtype, grade, TILs, Ki-67)	4.75	95% CI, 2.24–10.07; *p* < 0.001
Overall agreement	72.1%	Cohen’s κ = 0.45
Sensitivity of ypT0 for predicting ypN0	53.5%	
Specificity of ypT0 for predicting ypN0	91.9%	
Positive predictive value (PPV)	87.6%	13/105 (12.4%) had positive axillary nodes despite ypT0
Negative predictive value (NPV)	64.9%	80/228 (35.1%) had ypN0 despite residual breast tumor

Abbreviations: OR, odds ratio; CI, confidence interval; ypT0, breast pathological complete response; ypN0, axillary pathological complete response; ypN+, pathological regional lymph node involvement after neoadjuvant treatment; TILs, tumor-infiltrating lymphocytes; PPV, positive predictive value; NPV, negative predictive value.

**Table 10:** Association between breast and axillary pathological response according to molecular subtype.

Molecular Subtype	Breast Response	n	ypN0, n (%)
Luminal A-like	ypT0	2	0 (0.0%)
Luminal A-like	Residual breast tumor	55	13 (24.0%)
Luminal B-like	ypT0	14	9 (64.3%)
Luminal B-like	Residual breast tumor	109	31 (28.4%)
HER2-positive	ypT0	60	57 (95.0%)
HER2-positive	Residual breast tumor	31	17 (54.8%)
TNBC	ypT0	29	26 (89.7%)
TNBC	Residual breast tumor	33	19 (57.6%)

Abbreviations: ypT0, breast pathological complete response; ypN0, axillary pathological complete response; HER2, human epidermal growth factor receptor 2; TNBC, triple-negative breast cancer.

**Table 11:** Multivariable predictors of pathological complete response (ypT0N0).

Variable	Adjusted OR (95% CI)	*p*-Value
Age ≥50 years, Cohort A	0.55 (0.27–1.12)	0.099
Age ≥50 years, entire cohort	0.81 (0.46–1.41)	0.457
Nottingham grade 3, Cohort A	5.95 (2.57–13.77)	<0.001
Nottingham grade 3, entire cohort	6.40 (3.24–12.67)	<0.001
Ki-67 <20%, Cohort A	0.32 (0.09–1.18)	0.086
Ki-67 <20%, entire cohort	0.30 (0.10–0.91)	0.033
TILs <10%, Cohort A	0.30 (0.12–0.71)	0.007
TILs <10%, entire cohort	0.18 (0.08–0.38)	<0.001
DCIS <10%, Cohort A	2.34 (0.95–5.77)	0.065
DCIS <10%, entire cohort	2.32 (1.14–4.72)	0.020
Luminal B-like subtype (vs. Luminal A-like), Cohort A	Not estimable *	-
Luminal B-like subtype (vs. Luminal A-like), entire cohort	Not estimable *	-
HER2-positive subtype (vs. Luminal A-like), Cohort A	Not estimable *	-
HER2-positive subtype (vs. Luminal A-like), entire cohort	Not estimable *	-
TNBC subtype (vs. Luminal A-like), Cohort A	Not estimable *	-
TNBC subtype (vs. Luminal A-like), entire cohort	Not estimable *	-
cT3–4 (vs. cT1–2), Cohort A	0.37 (0.17–0.79)	0.010
cT3–4 (vs. cT1–2), entire cohort	0.45 (0.25–0.81)	0.008

Abbreviations: OR, odds ratio; CI, confidence interval; ypT0N0, pathological complete response in both the breast and axillary lymph nodes; TILs, tumor-infiltrating lymphocytes; DCIS, ductal carcinoma in situ; HER2, human epidermal growth factor receptor 2; TNBC, triple-negative breast cancer. Model estimated with Firth’s penalized likelihood. * No Luminal A-like tumor achieved ypT0N0; OR relative to Luminal A-like cannot be estimated (quasi-complete separation).

**Table 12:** Overall survival according to pathological response and molecular subtype. Median follow-up: 38.7 months (95% CI, 35.2–42.1); 26 deaths and 21 recurrences.

Group	n	Events	3-Year OS, % (95% CI)	5-Year OS, % (95% CI)
Overall pCR (log-rank *p* = 0.278)				
pCR (ypT0N0)	92	5	94.8 (89.9–99.8)	92.4 (85.5–99.2)
No pCR	241	21	93.4 (89.9–96.9)	86.0 (79.6–92.3)
Axillary pCR (log-rank *p* = 0.952)				
ypN0	172	14	92.8 (88.5–97.2)	89.0 (83.1–95.0)
ypN+	161	12	95.0 (91.3–98.6)	86.3 (78.0–94.5)
Breast pCR (log-rank *p* = 0.282)				
ypT0	105	6	95.5 (91.1–99.9)	90.7 (83.0–98.4)
Residual breast tumor	228	20	93.0 (89.4–96.7)	86.4 (80.0–92.7)
Molecular subtype (log-rank *p* = 0.032)				
Luminal A-like	57	6	96.0 (90.6–100.0)	74.9 (54.8–95.0)
Luminal B-like	123	7	95.5 (91.6–99.4)	91.4 (84.8–98.1)
HER2-positive	91	4	95.8 (91.0–100.0)	93.1 (86.2–100.0)
TNBC	62	9	84.5 (73.7–95.4)	81.0 (68.6–93.4)

Abbreviations: OS, overall survival; pCR, pathological complete response; ypT0N0, pathological complete response in the breast and axillary lymph nodes; ypT0, breast pathological complete response; ypN0, axillary pathological complete response; HER2, human epidermal growth factor receptor 2; TNBC, triple-negative breast cancer; CI, confidence interval.

**Table 13:** Disease-free survival according to pathological response and molecular subtype.

Group	n	Events	3-Year DFS, % (95% CI)	5-Year DFS, % (95% CI)
Overall pCR (log-rank *p* = 0.040)				
pCR (ypT0N0)	92	5	94.8 (89.9–99.8)	92.4 (85.5–99.2)
No pCR	241	31	90.1 (85.8–94.4)	79.0 (71.7–86.3)
Axillary pCR (log-rank *p* = 0.098)				
ypN0	172	15	92.1 (87.5–96.6)	86.6 (79.7–93.4)
ypN+	161	21	90.8 (85.6–95.9)	78.4 (69.1–87.6)
Breast pCR (log-rank *p* = 0.075)				
ypT0	105	7	94.1 (89.0–99.2)	89.3 (81.1–97.4)
Residual breast tumor	228	29	90.2 (85.7–94.6)	79.6 (72.3–87.0)
Molecular subtype (log-rank *p* = 0.153)				
Luminal A-like	57	7	96.1 (90.7–100.0)	73.1 (54.6–91.5)
Luminal B-like	123	13	92.1 (86.8–97.5)	85.0 (76.6–93.4)
HER2-positive	91	6	92.4 (85.8–98.9)	89.7 (81.4–97.9)
TNBC	62	10	83.8 (73.3–94.3)	74.1 (58.0–90.2)

Abbreviations: DFS, disease-free survival; pCR, pathological complete response; ypT0N0, pathological complete response in the breast and axillary lymph nodes; ypT0, breast pathological complete response; ypN0, axillary pathological complete response; HER2, human epidermal growth factor receptor 2; TNBC, triple-negative breast cancer; CI, confidence interval.

**Table 14:** Univariable Cox regression analysis of overall survival and disease-free survival.

Variable	Crude HR for OS (95% CI)	*p*-Value (OS)	Crude HR for DFS (95% CI)	*p*-Value (DFS)
Overall pCR (ypT0N0)	0.59 (0.22–1.56)	0.284	0.38 (0.15–0.99)	0.047
ypN0	0.98 (0.45–2.11)	0.951	0.57 (0.30–1.12)	0.102
ypT0	0.61 (0.24–1.52)	0.287	0.48 (0.21–1.10)	0.082
Age ≥50 years	2.05 (0.77–5.44)	0.149	1.50 (0.70–3.19)	0.294
Nottingham grade 3	1.14 (0.53–2.49)	0.733	0.99 (0.51–1.94)	0.984
Ki-67 <20%	0.90 (0.38–2.14)	0.808	0.68 (0.31–1.48)	0.329
TILs <10%	1.04 (0.48–2.28)	0.917	1.29 (0.67–2.48)	0.452
DCIS <10%	1.69 (0.58–4.90)	0.336	1.53 (0.64–3.67)	0.343
ER/PR positive	0.68 (0.31–1.50)	0.338	0.84 (0.42–1.68)	0.625
HER2-positive	0.43 (0.15–1.26)	0.125	0.47 (0.20–1.14)	0.094
cT3–4	2.09 (0.97–4.52)	0.061	1.78 (0.92–3.46)	0.087
≥4 residual positive lymph nodes	2.50 (1.09–5.76)	0.031	2.55 (1.26–5.20)	0.010

Abbreviations: HR, hazard ratio; OS, overall survival; DFS, disease-free survival; pCR, pathological complete response; ypT0N0, pathological complete response in the breast and axillary lymph nodes; ypT0, breast pathological complete response; ypN0, axillary pathological complete response; TILs, tumor-infiltrating lymphocytes; DCIS, ductal carcinoma in situ; ER, estrogen receptor; PR, progesterone receptor; HER2, human epidermal growth factor receptor 2; CI, confidence interval.

**Table 15:** Multivariable Cox regression analysis of overall survival and disease-free survival. Multivariable analysis was restricted to three predictors because of the limited number of events (26 deaths for OS and 36 events for DFS).

Variable	Adjusted HR for OS (95% CI)	*p*-Value (OS)	Adjusted HR for DFS (95% CI)	*p*-Value (DFS)
Overall pCR (ypT0N0)	0.54 (0.18–1.61)	0.268	0.35 (0.13–0.99)	0.048
≥4 residual positive lymph nodes	2.46 (1.01–5.98)	0.046	2.27 (1.08–4.78)	0.031
ER/PR positive	0.46 (0.20–1.10)	0.080	0.51 (0.24–1.07)	0.074

Abbreviations: HR, hazard ratio; OS, overall survival; DFS, disease-free survival; pCR, pathological complete response; ypT0N0, pathological complete response in the breast and axillary lymph nodes; ER, estrogen receptor; PR, progesterone receptor; CI, confidence interval.

## Data Availability

The original contributions presented in this study are included in the article. Further inquiries can be directed to the corresponding authors.
